# Membrane protease prostasin promotes insulin secretion by regulating the epidermal growth factor receptor pathway

**DOI:** 10.1038/s41598-023-36326-7

**Published:** 2023-06-05

**Authors:** Toshihisa Ishii, Yoshikazu Miyasato, Masashi Ichijo, Kohei Uchimura, Fumihiko Furuya

**Affiliations:** 1 Division of Nephrology, University of Yamanashi, Yamanashi, 409-3898 Japan; 2 Department of Nephrology, Kumamoto University Graduate School of Medical Sciences, Kumamoto, 860-8556 Japan; 3 Department of Diabetes and Endocrinology, Matsumoto National Hospital, Matsumoto, Japan; 4 Department of Thyroid and Endocrinology, Fukushima Medical University, Fukushima, Japan

**Keywords:** Cell biology, Molecular biology

## Abstract

Prostasin (PRSS8) is a serine protease that metabolizes and moderates the effect of specific substrates. Epidermal growth factor receptor (EGFR), which modulates insulin secretion and pancreatic β-cell proliferation, is regulated via proteolytic shedding by PRSS8. We first detected PRSS8 expression in β-cells of pancreatic islets of mice. To better understand the molecular processes involved in PRSS8-associated insulin secretion, pancreatic β-cell-specific PRSS8 knockout (βKO) and PRSS8-overexpressing (βTG) male mice were generated. We found that glucose intolerance and reduction in glucose-stimulated insulin secretion developed in βKO mice compared with the control subjects. A higher response to glucose was noted in islets retrieved from βTG mice. Erlotinib, a specific blocker of EGFR, blocks EGF- and glucose-stimulated secretion of insulin among MIN6 cells, and glucose improves EGF release from β-cells. After silencing PRSS8 in MIN6 cells, glucose-stimulated insulin secretion decreased, and EGFR signaling was impaired. Conversely, overexpression of PRSS8 in MIN6 cells induced higher concentrations of both basal and glucose-stimulated insulin secretion and increased phospho-EGFR concentrations. Furthermore, short-term exposure to glucose improved the concentration of endogenous PRSS8 in MIN6 cells through inhibition of intracellular degradation. These findings suggest that PRSS8 is involved in glucose-dependent physiological regulation of insulin secretion via the EGF–EGFR signaling pathway in pancreatic β-cells.

## Introduction

Diabetes is a chronic metabolic condition manifesting as chronic hyperglycemia attributable to abnormal insulin secretion or insulin resistance. Functional defects and deficit of β-cell mass results in reduced insulin secretion^1^. Depletion of endogenous insulin secretion limits therapeutic options and makes it difficult to control diabetic complications. Human islets possess limited regenerative ability, and there is no established therapy to restore depleted β-cell mass^2^. The main strategies to replenish β-cells are the production and transplantation of new β-cells using the human pluripotent stem cells and the induction of endogenous regeneration. To prevent diabetic complications or cardiovascular disease, it is an urgent priority to develop therapeutic strategies to maintain and preserve β-cells.

Epidermal growth factor (EGF) is a crucial growth agent responsible for the proliferation of fibroblasts and epithelial cells^3^, and it is also a modulator that induces acrosomal exocytosis and the expression of many hormones^4,5^. The epidermal growth factor receptor (EGFR) is expressed in pancreatic β-cells, and the long-term effects of EGFR signaling are associated with proliferation and differentiation. Mice lacking EGFR develop physiologically abnormal islets^6^, indicating that EGFR signaling is essential for proper pancreatic development. EGFR signaling is also required for murine β-cell proliferation in reaction to a high fat diet^7^. Short-term EGFR signaling affects insulin secretion. Intravenous glucose administration has been found to elevate blood EGF levels in vivo. After 2–4 min of incubation with 1–100-nM EGF, ligand-bound EGFR induced Ca^2+^ influx, phospholipase D2 (PLD2) activation, and insulin secretion from pancreatic β-cells in a concentration-dependent process, indicating that EGF-induced insulin secretion is related to glucose-stimulated insulin secretion^8^.

PRSS8, which was first detected in seminal fluid, is a GPI-anchored or secreted serine protease with a molecular weight of 40 kDa^9^. PRSS8 is expressed in various organs, including the prostate gland, kidney, lung, colon, liver, and pancreas^10^. However, the role of PRSS8 in most of these organs is unknown. Serine proteases play critical roles in diverse processes via cleavage of their substrates. One of the most well-known functions of PRSS8 is the activation of epithelial Na channels (ENaCs). PRSS8 and furin cleave ENaCs to regulate sodium transport^8^. Uchimura et al. demonstrated that decreased hepatic PRSS8 expression is involved in the onset of diabetes due to satiety and obesity, and PRSS8 regulates insulin resistance in the liver via cleavage of TLR4^11^. Interestingly, the levels of PRSS8 were reduced in glucose-intolerant patients in this study.

EGFR is another substrate of PRSS8 and is activated by its cleavage, although its significance is unknown^12^. This study aim was to reveal the localization and function of PRSS8 in pancreatic islets. We hypothesized that EGFR is a substrate of PRSS8 in pancreatic β-cells and that PRSS8 modulates insulin secretion via EGF–EGFR signaling. Our study provides new insights into the impact of EGFR signaling on glucose-induced insulin secretion.

## Results

### PRSS8 is expressed in the membranes of pancreatic β-cells

**Figure 1:**
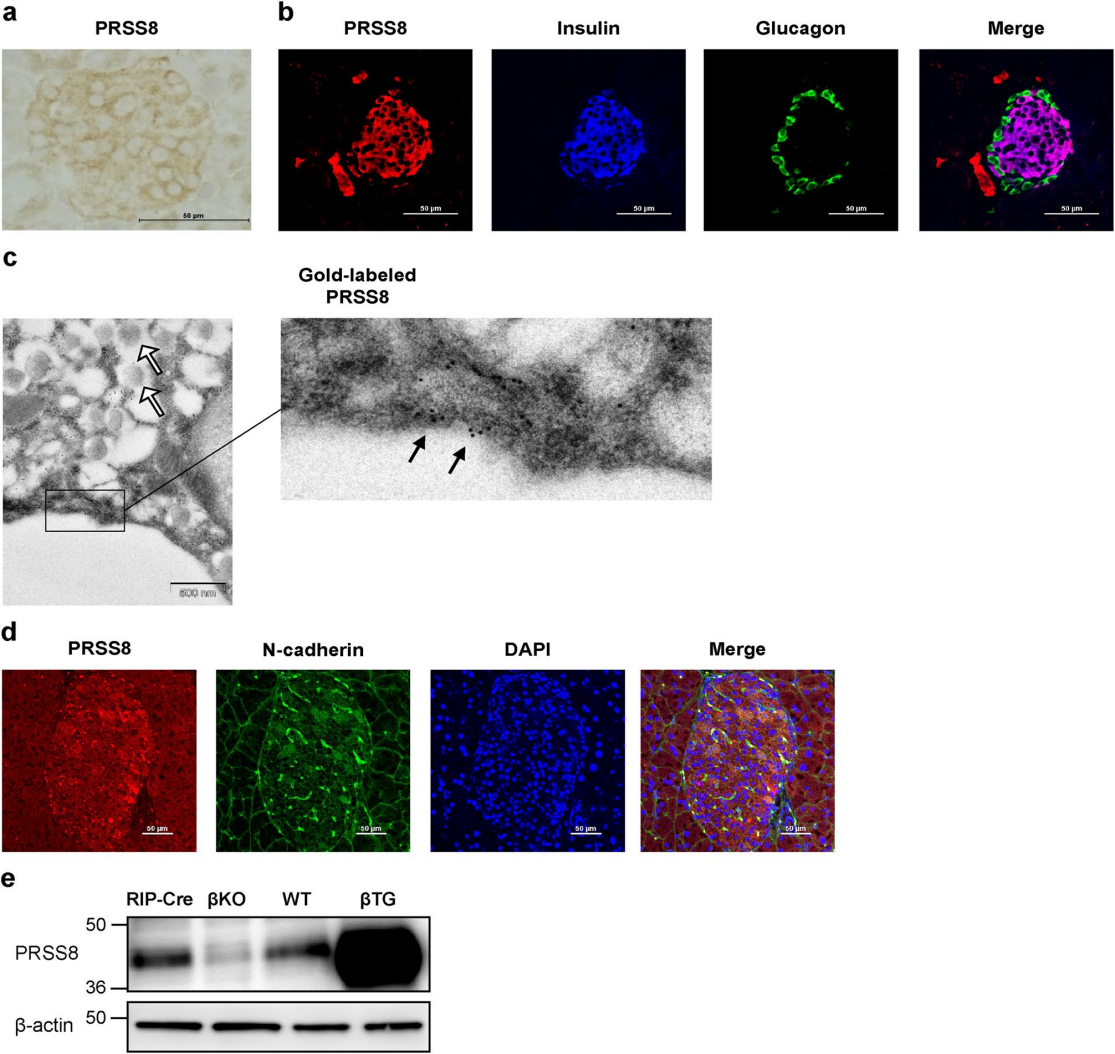
Prostasin (PRSS8) expression in pancreatic β-cells. (**a**) DAB staining of islets isolated from 8-week-old wild-type (WT) mice. (**b**) Immunofluorescent staining of islets from WT mice at 8 weeks of age. Red: PRSS8 (BD Biosciences); blue: insulin; green: glucagon. (**c**) Immunoelectron microscopy of β-cells from WT mice at 8 weeks of age. Black arrows denote PRSS8 labeled with colloidal gold. White arrows denote insulin granules. (**d**) Immunofluorescent staining of islets from 8-week-old WT mice. Red: PRSS8 (PA5-27,977, Thermo Fisher Scientific); green: N-cadherin; blue: DAPI for nuclei. (**e**) Western blotting for PRSS8 and β-actin using the protein lysates of isolated islets (Supplementary Fig. S1). RIP-Cre: rat insulin promoter-driven Cre transgenic mice; βKO: PRSS8-knockout mice; βTG: PRSS8-overexpressing mice.

Immunohistochemical analysis indicated that PRSS8 was expressed in islets and exocrine cells (Fig. 1a). PRSS8 was co-localized with insulin-positive cells in the islets (Fig. 1b). Immunoelectron microscopy showed that PRSS8 was expressed in the cell membrane and endoplasmic reticulum membrane along with insulin granules (Fig. 1c). These results suggest that PRSS8 is expressed in pancreatic β-cells. PRSS8 also co-stained with N-cadherin, a cell membrane protein^13^ (Fig. 1d). To analyze the function of PRSS8 in pancreatic β-cells, we developed β-cell-specific PRSS8 knockout (βKO) and PRSS8-overexpressing (βTG) mice. In the western blotting analyses (Fig. 1e and Supplementary Fig. S1), the upper band indicated the zymogen form of PRSS8, and the lower band indicated the active form^14^. The abundance of PRSS8 in the islets was decreased in βKO mice and enhanced in βTG mice compared with the respective control levels. βKO mice are RIP-Cre^+/−^PRSS8^lox/lox^; therefore PRSS8 expression was not completely deleted in these mice. PRSS8 expression was not significantly different between wild-type (WT) and RIP-Cre mice.

### Endogenous PRSS8 regulates insulin secretion in pancreatic β-cells

**Figure 2:**
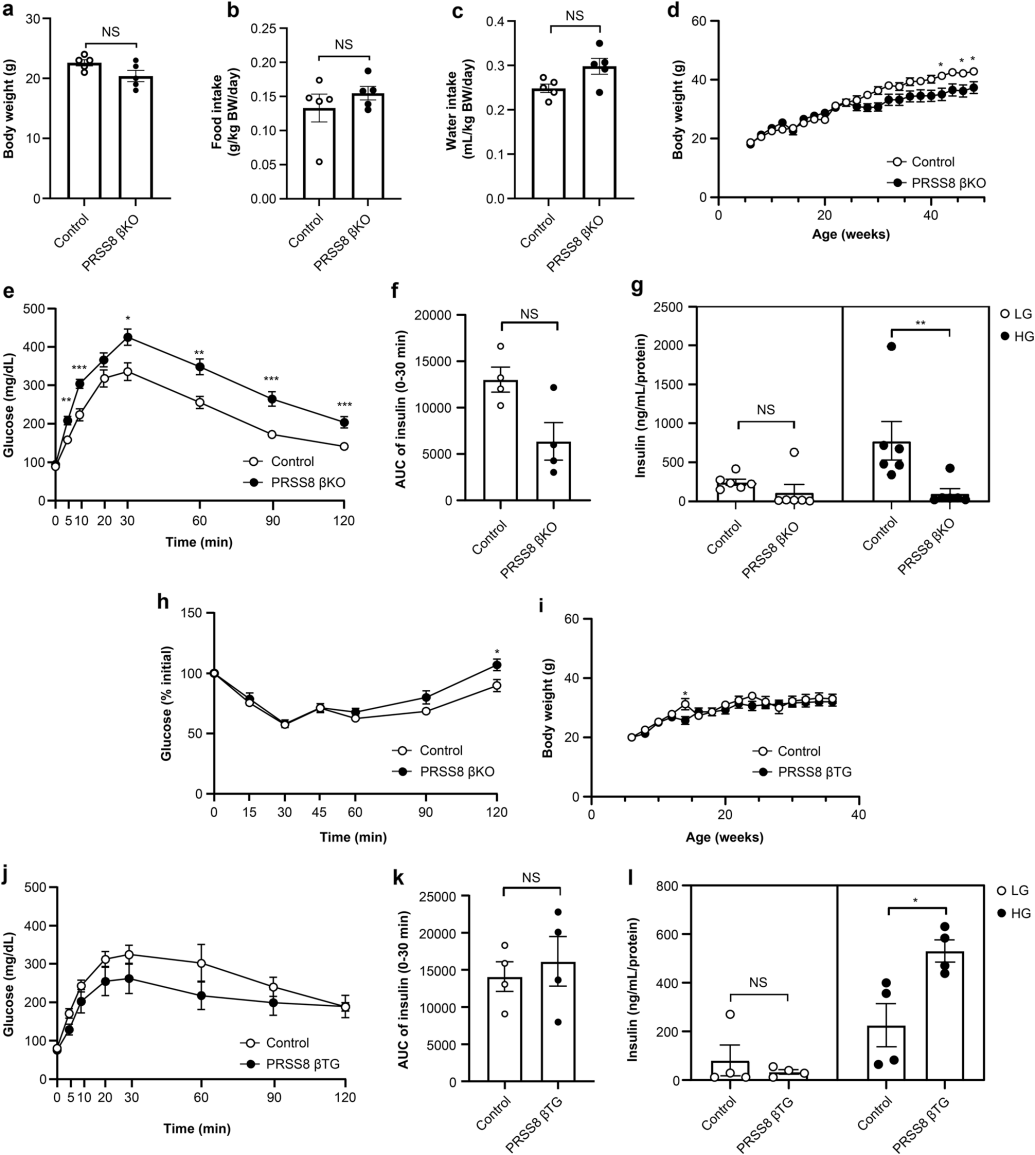
Glucose metabolic parameters in prostasin (PRSS8)-knockout (βKO) and PRSS8-overexpressing (βTG) mice. (**a**–**c**) Body weight (BW) (**g**), food intake (g/kg BW), and water intake (mL/kg BW) in 7–8-week-old male mice (n = 5/group). (**d**, **i**) BW curve. Rat insulin promoter-driven Cre transgenic (RIP-Cre) and βKO in (D) (n = 3–20). Wild-type and PRSS8-βTG mice in (I) (n = 2–13). (**e**, **j**) The intraperitoneal glucose tolerance test (IPGTT) in mice. RIP-Cre (n = 17) and PRSS8 βKO (n = 18) in (**e**). Wild-type (WT, n = 8) and PRSS8-βTG mice (n = 7) in (**j**). Blood glucose levels were measured 0, 5, 10, 20, 30, 60, 90, and 120 min after a glucose challenge at 1.5 g/kg BW. (**f**, **k**) The area under the curve (AUC) of insulin secretion at 0, 10, 20, and 30 min after a glucose challenge at 1.5 g glucose/kg BW (n = 4/group). (**g**, **l**) Islets isolated from RIP-Cre (n = 6), βKO (n = 6), WT (n = 4), and βTG (n = 4) mice were stimulated with 3 mM (low glucose [LG]) or 20 mM glucose (high glucose [HG]) for 60 min. (**h**) Insulin tolerance test. Blood glucose levels at 0, 15, 30, 45, 60, 90, and 120 min after an insulin challenge at 1.0 U/kg BW (n = 8/group). All data are presented as the mean ± SEM (error bars). NS, not significant; *P < 0.05; **P < 0.01; ***P < 0.001.

To investigate PRSS8 role within pancreatic β-cells, we examined metabolic parameters in βKO and βTG mice. The 7–8-week-old control and βKO mice did not significantly differ in terms of their body weight, food intake, and water intake (Fig. 2a–c). Nevertheless, aging βKO mice tended to have lower body weights (Fig. 2d). In the intraperitoneal glucose tolerance test (IPGTT), βKO mice showed sustained increased glucose levels in the early phase (5–120 min) after glucose injection (Fig. 2e). The area under the curve (AUC) of insulin secretion at 0–30 min in βKO mice was small (Fig. 2f, *P* = 0.08). To assess insulin secretion in pancreatic islets, we isolated dispersed islets from mice and measured insulin release ex vivo. βKO islets treated with high glucose displayed significantly lower insulin secretion (Fig. 2g). Because both insulin insufficiency and insulin resistance can manifest with glucose intolerance, we carried out the insulin tolerance test (ITT) to differentiate the two scenarios. There was no sufficient variation in insulin sensitivity between control and βKO mice, indicating that the glucose intolerance may not be attributable to insulin resistance (Fig. 2h). Conversely, no significant differences were detected in body weights, IPGTT results, and the AUC of insulin secretion over time between control and βTG mice (Fig. 2i,j,k). In contrast to this result, βTG mice exhibited significantly greater insulin secretion than controls ex vivo (Fig. 2l).

### EGF–EGFR signaling amplifies glucose-stimulated insulin secretion

**Figure 3:**
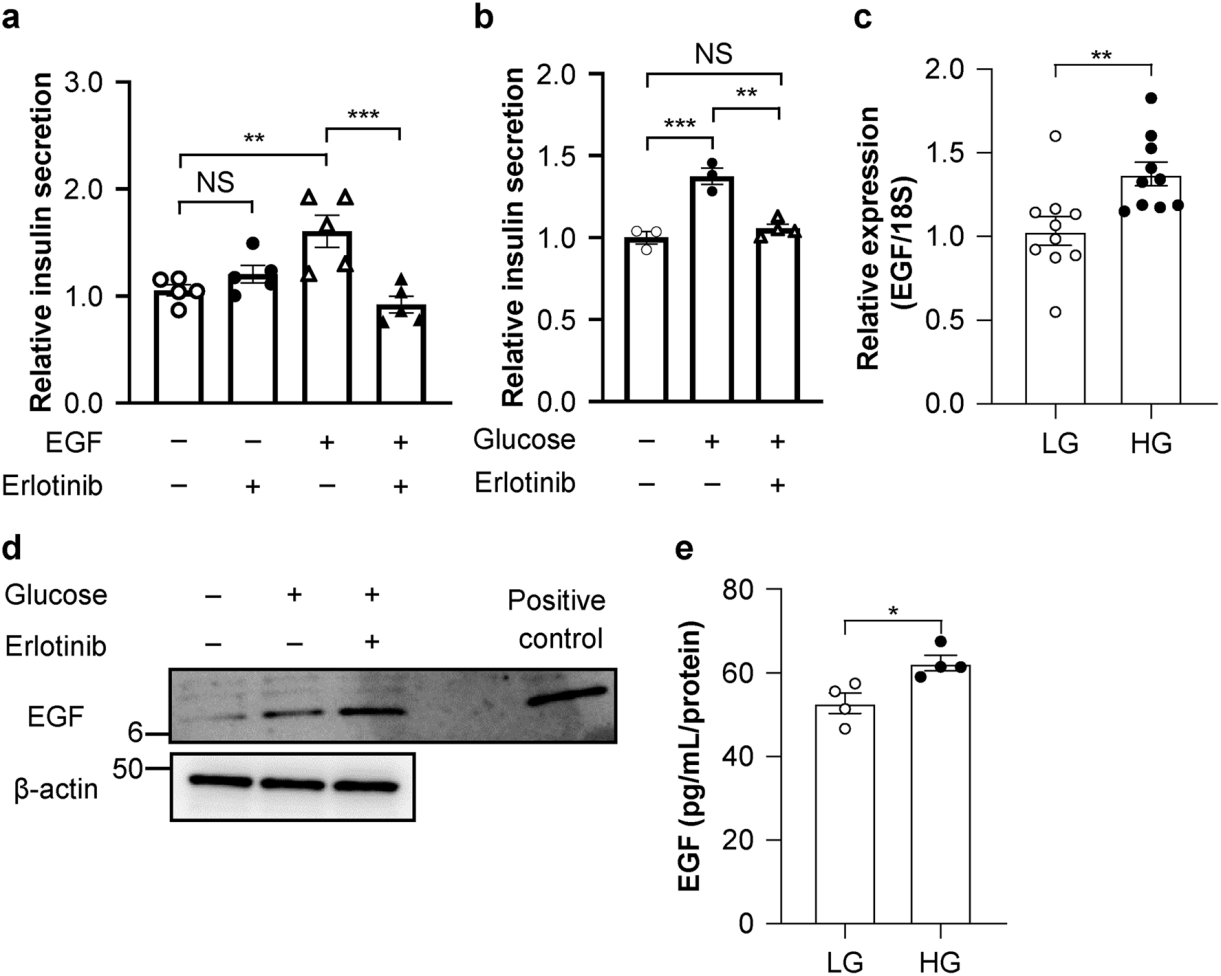
Inhibition of epidermal growth factor receptor (EGFR) signaling impairs glucose-stimulated insulin secretion (GSIS). (**a**) Insulin secretion in MIN6 cells treated with 50 nM epidermal growth factor (EGF) and/or 30 nM erlotinib for 60 min (n = 6/group). (**b**) Insulin secretion in MIN6 cells treated with 20 mM glucose and/or 30 nM erlotinib (n = 3–4/group). (**c**) mRNA expression of EGF after glucose administration (n = 10/group). Low glucose (LG), 3 mM glucose; high glucose (HG), 20 mM glucose. (**d**) Western blotting for EGF following treatment with 20 mM glucose and/or 30 nM erlotinib (Supplementary Fig. S2). (**e**) The concentration of EGF in the supernatant after glucose administration (n = 4/group). All data are presented as the mean ± SEM (error bars). NS, not significant; *P < 0.05; **P < 0.01; ***P < 0.001.

As preliminary experiments, we explored the role of EGF–EGFR signaling to glucose-stimulated insulin secretion (GSIS) in MIN6 cells. Consistent with a previous report^8^, EGF treatment increased insulin secretion (Fig. 3a), which was suppressed by the selective EGFR tyrosine kinase inhibitor, erlotinib. Importantly, erlotinib suppressed GSIS in the absence of EGF treatment (Fig. 3b). These results suggest that glucose induces EGF–EGFR signaling in vitro. To clarify whether EGF is required for insulin secretion in response to EGFR signaling, we investigated the behavior of EGF. High glucose increased the mRNA expression, protein expression, and supernatant concentrations of EGF (Fig. 3c–e, Supplementary Fig. S2).

### PRSS8 promotes insulin secretion via EGFR activation

**Figure 4:**
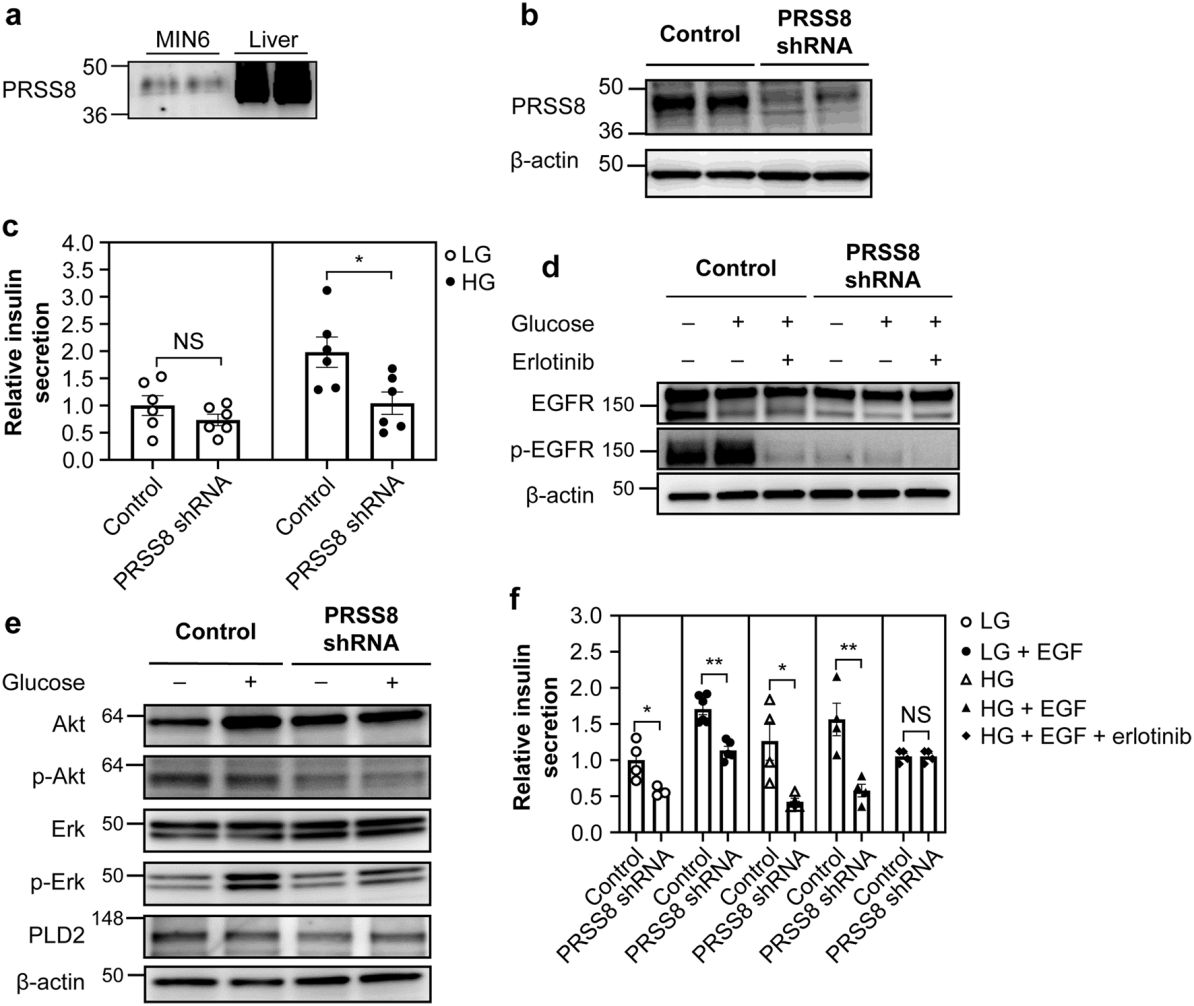
Prostasin (PRSS8) deficiency impairs glucose-stimulated insulin secretion (GSIS) and epidermal growth factor receptor (EGFR) activation. (**a**) Western blotting for PRSS8 in MIN6 cells. Liver protein lysates were used as a positive control. (**b**) Western blotting for PRSS8 following PRSS8 depletion (KD) (Supplementary Fig. S3). (**c**) GSIS in control and PRSS8-depleted MIN6 cells for 60 min (n = 6/group). Low glucose (LG), 3 mM glucose; high glucose (HG), 20 mM glucose (Supplementary Fig. S4). (**d**) Western blotting for EGFR, p-EGFR, and β-actin in PRSS8-depleted cells (Supplementary Fig. S6). (**e**) Western blotting for Akt, p-Akt, Erk, p-Erk, phospholipase D2 (PLD2), and β-actin in PRSS8-depleted cells (Supplementary Fig. S7). (**f**) Insulin secretion in PRSS8-depleted cells following treatment with 50 nM epidermal growth factor (EGF) and 30 nM erlotinib (n = 4/group). LG, 3 mM glucose; HG, 20 mM glucose (Supplementary Fig. S9). All data are presented as the mean ± SEM (error bars). NS, not significant; *P < 0.05; **P < 0.01.

MIN6 cells also expressed PRSS8 (Fig. 4a). To investigate the contribution of PRSS8 to insulin secretion, we evaluated insulin secretion in MIN6 cells following PRSS8 depletion or overexpression. PRSS8 depletion (Fig. 4b, Supplementary Fig. S3) significantly reduced insulin secretion (Fig. 4c, Supplementary Fig. S4). Various segments of EGFR are detected by the shedding of PRSS8 and Matriptase. In a previous report^12^, these segments were detected as a band near 170 kDa, and modification with PRSS8 also detected bands at 135 and 110 kDa. In this study, exposure to high glucose for 10 min induced EGFR activation in the control MIN6 cells, and phosphorylation of the latter was detected in the 170 kDa and 135 kDa bands (Fig. 4d). Note that both bands were detected by treatment with EGF (Supplementary Fig. S5), suggesting that EGFR is phosphorylated by glucose in a similar manner to EGF. Phosphorylation of EGFR by glucose was canceled by erlotinib (Fig. 4d, Supplementary Fig. S6). However, PRSS8 depletion strongly suppressed EGFR activation in response to high glucose. In addition, PRSS8 depletion resulted in reduced p-Akt, p-Erk, and PLD2 expression when assessed in relation to the control levels (Fig. 4e, Supplementary Fig. S7). EGFR phosphorylation induced by EGF exposure was also suppressed in PRSS8 depletion (Supplementary Fig. S8). Consistent with these results, PRSS8 depletion suppressed insulin secretion in reaction to glucose and EGF (Fig. 4f, Supplementary Fig. S9). These differences were canceled by erlotinib, suggesting that PRSS8 regulates insulin secretion via EGFR activation.

**Figure 5:**
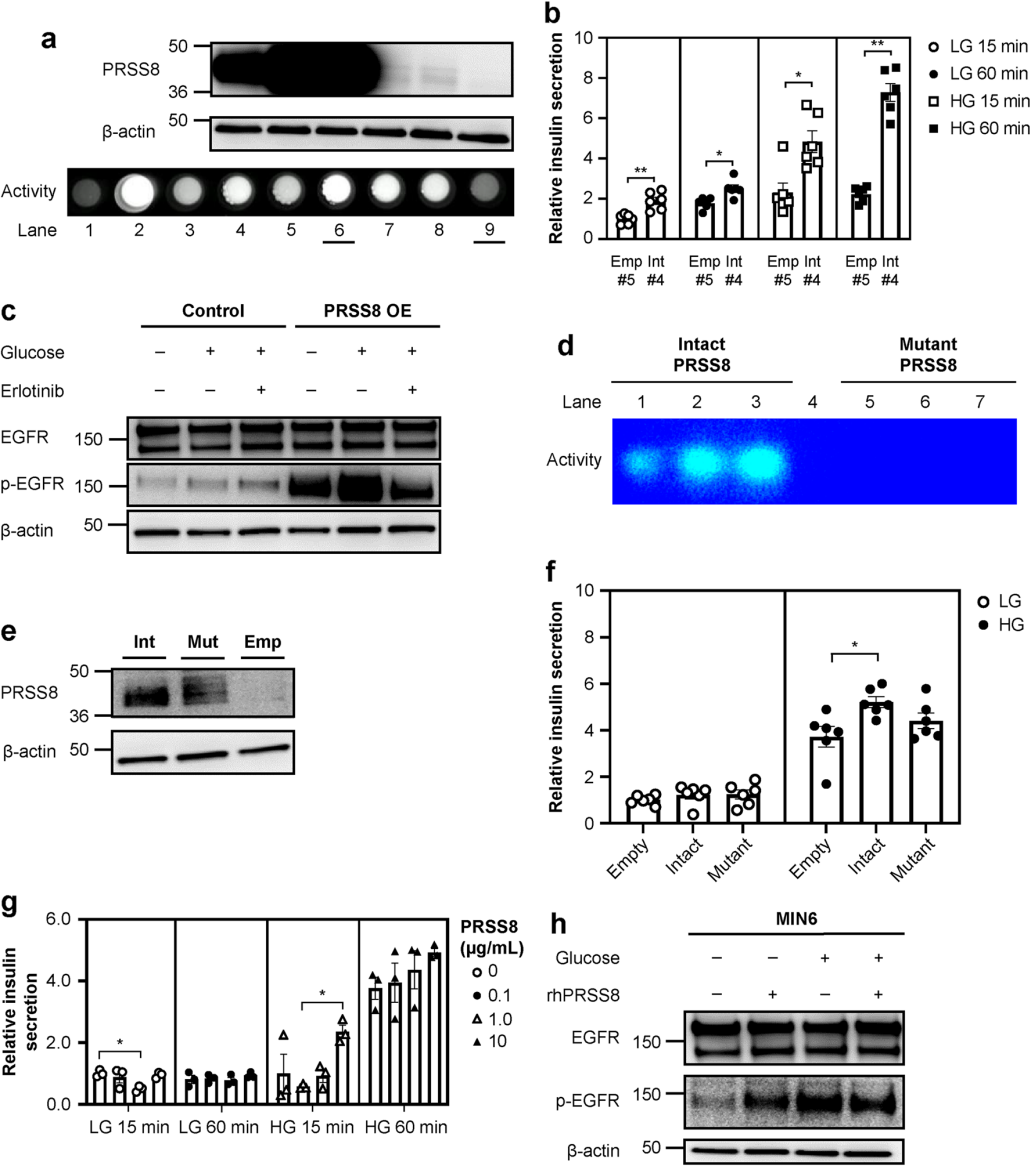
Prostasin (PRSS8) overexpression promotes insulin secretion and epidermal growth factor receptor (EGFR) activation. (**a**) Western blotting for PRSS8 and β-actin in control and stable PRSS8-overexpressing (OE) MIN6 cell lines (Supplementary Fig. S10). Activity was evaluated by zymography. Lane 1, negative control; Lane 2, positive control (trypsin, 1 mg); Lane 3, recombinant human PRSS8 (rhPRSS8) 1 mg; Lane 4, Intact #2; Lane 5, Intact #3; Lane 6, Intact #4; Lane 7, Empty #2; Lane 8, Empty #3; and Lane 9, Empty #5. The cell lines presented in Lanes 6 and 9 were used for the following experiments. (**b**) Insulin secretion in PRSS8 OE cells for 15 and 60 min (n = 6/group). Low glucose (LG), 3 mM glucose; high glucose (HG), 20 mM glucose. (**c**) Western blotting for EGFR, p-EGFR, and β-actin in PRSS8 OE MIN6 cells treated with 20 mM glucose and erlotinib (Supplementary Fig. S10). (**d**) The activity of intact and mutant (loss of function mutation) PRSS8 was evaluated by zymography in HEK-293 cells. (**e**) Western blotting for PRSS8 and β-actin in MIN6 cells transfected by intact PRSS8, mutant PRSS8, and empty vector (Supplementary Fig. S13). (**f**) Glucose-stimulated insulin secretion in MIN6 cells transiently transfected with the empty vector, intact PRSS8, and mutant PRSS8 for 60 min (n = 6/group). LG, 3 mM; HG, 20 mM. (**g**) Insulin secretion in MIN6 cells treated with rhPRSS8 for 15 and 60 min. The concentration of rhPRSS8 is indicated. (**h**) Western blotting for EGFR, p-EGFR, and β-actin in MIN6 cells treated with 20 mM glucose and/or 1.0 mg/mL rhPRSS8 (Supplementary Fig. S14). All data are presented as the mean ± SEM (error bars). NS, not significant; *P < 0.05; **P < 0.01; ***P < 0.001.

We next examined insulin secretion and EGFR activation in PRSS8-overexpressing cells. The pair of monoclonal cell lines with the largest contrast in PRSS8 expression and activity was used for comparison (Fig. 5a, Supplementary Fig. S10). PRSS8 overexpression significantly increased insulin secretion from the early phase compared with controls after empty vector transfection (Fig. 5b). Consistent with these results, PRSS8 overexpression enhanced EGFR activation by both glucose and EGF (Fig. 5c, Supplementary Figs. S11, S12). Further, to investigate whether the protease activity of PRSS8 affects insulin secretion, we used cells expressing mutant PRSS8, in which its activity was completely abrogated (Fig. 5d,e, Supplementary Fig. S13). Insulin secretion in mutant PRSS8-overexpressing cells was not increased compared to the control (Fig. 5f). To demonstrate whether PRSS8 regulates insulin secretion via the short-term effect of EGFR, MIN6 cells were treated with recombinant human PRSS8 (rhPRSS8), which is the active form. rhPRSS8 significantly increased insulin secretion after 15 min of treatment and tended to increase insulin secretion in a concentration-dependent manner (Fig. 5g). EGFR activation was observed following treatment with glucose, rhPRSS8, or both (Fig. 5h, Supplementary Fig. S14).

### Endogenous expression of PRSS8 is regulated by blood glucose concentrations

**Figure 6:**
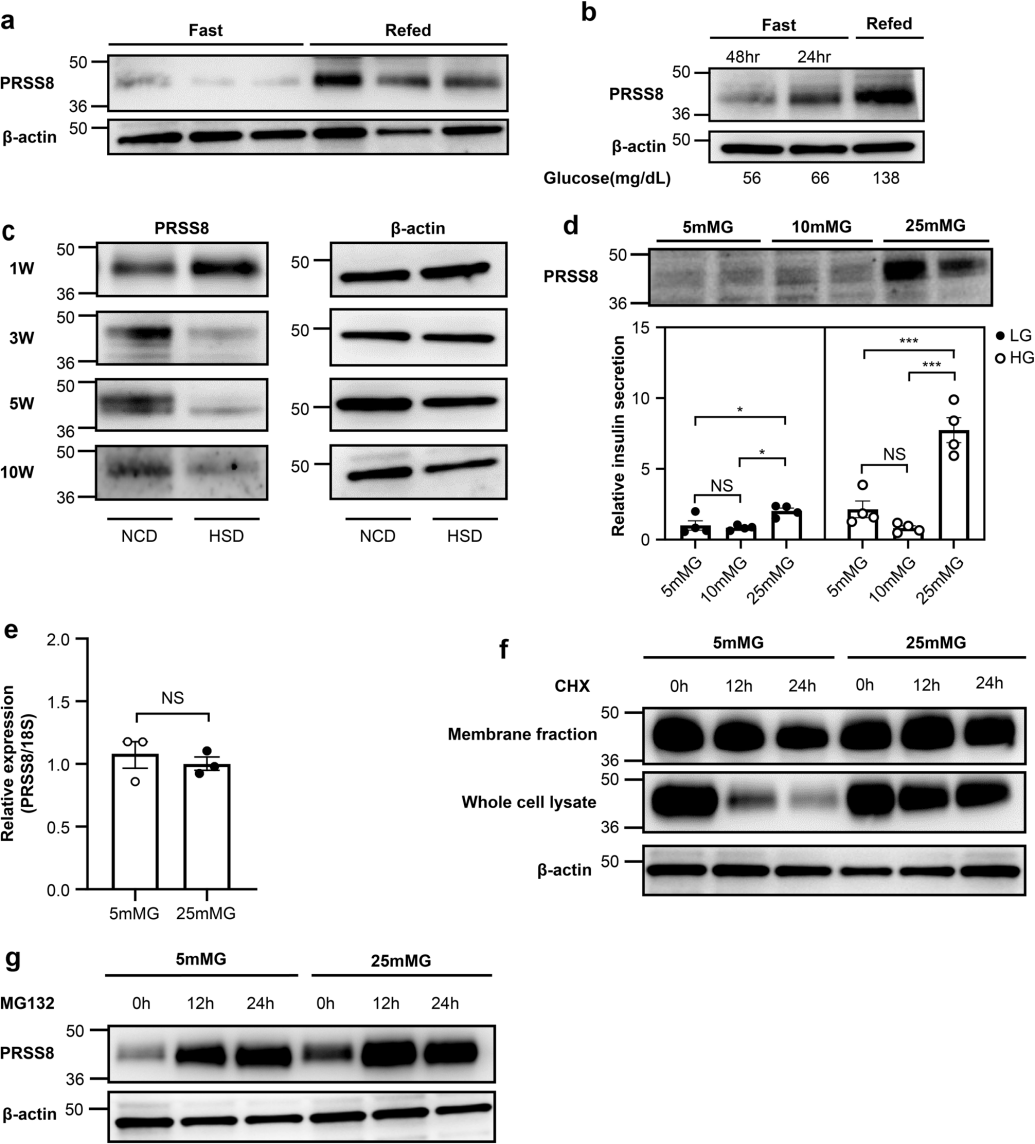
Glucose regulates prostasin (PRSS8) expression. (**a**, **b**) Western blotting for PRSS8 and β-actin in islets isolated from WT mice under fasting and refeeding conditions. Blood glucose levels under fasting for 24 or 48 h and refeeding are presented in B (Supplementary Figs. S15, S16). (**c**) Western blotting for PRSS8 and β-actin in islets isolated from WT mice fed a normal control diet (NCD) or high-sucrose diet (HSD, 67 kcal%) at the indicated weeks (Supplementary Fig. S17). (**d**) Western blotting for PRSS8 and insulin secretion in MIN6 cells pretreated with 5, 10, or 25 mM glucose (n = 4/group) (Supplementary Fig. S18). Low glucose (LG), 3 mM glucose; high glucose (HG), 20 mM glucose. (**e**) mRNA expression of PRSS8 in MIN6 cells treated with 5 or 25 mM glucose (n = 3/group). (**f**) Time course of PRSS8 expression was analyzed by western blotting in the whole-cell lysate and membrane fraction of PRSS8 OE MIN6 cells treated with 40 mM cycloheximide (CHX) under 5 or 25 mM glucose (Supplementary Fig. S19). (**g**) Time course of PRSS8 expression analyzed by western blotting in the whole-cell lysates of PRSS8 OE MIN6 cells treated with 10 µM MG132 under 5 or 25 mM glucose (Supplementary Fig. S20). All data are presented as the mean ± SEM (error bars). NS, not significant; *P < 0.05; **P < 0.01.

Next, we investigated the regulation of PRSS8 expression. PRSS8 expression in isolated islets from WT mice under refeeding conditions was markedly higher than that in mice under fasting conditions (Fig. 6a, Supplementary Fig. S15). PRSS8 expression was reduced in mice with lower blood glucose levels following prolonged fasting (Fig. 6b, Supplementary Fig. S16). Therefore, we hypothesized that PRSS8 expression was dependent on the glucose concentration. Conversely, mice fed a high-sucrose diet (HSD) for 3, 5, or 10 weeks exhibited lower PRSS8 expression than mice fed a normal control diet (NCD) (Fig. 6c, Supplementary Fig. S17).

Next, we examined the behavior of PRSS8 expression in response to glucose in vitro. MIN6 cells pre-incubated with 25 mM glucose displayed higher PRSS8 expression and insulin secretion than cells pre-incubated with low glucose (Fig. 6d, Supplementary Fig. S18). PRSS8 protein concentration improved in reaction to glucose without a change in mRNA levels (Fig. 6e). To observe a clear change, we examined PRSS8 degradation in PRSS8-overexpressing MIN6 cells. After treatment with cycloheximide (CHX), a protein synthesis inhibitor, PRSS8 degradation was suppressed under high-glucose conditions (Fig. 6f, Supplementary Fig. S19). The extent of reduction of PRSS8 levels in whole-cell lysates was greater than that in the membrane fraction under low-glucose conditions, suggesting that intracellular PRSS8 was degraded. Supporting this result, no differences in PRSS8 synthesis were observed between low-glucose and high-glucose conditions after treatment with MG132, a proteasome inhibitor (Fig. 6g, Supplementary Fig. S20). These findings indicate that PRSS8 secretion is modulated by glucose via degradation.

## Discussion

The findings of the present study show the crucial function of PRSS8 in GSIS in mouse β-cells. The expression or activity of PRSS8 in β-cells affected insulin secretion. In particular, PRSS8 overexpression or rhPRSS8 treatment induced rapid insulin release after glucose loading, suggesting that PRSS8 contributes to the first phase of insulin secretion^15^.

Mechanistically, our observations revealed that PRSS8 promotes insulin secretion by activating EGFR and its downstream signaling. A previous study reported insulin secretion via EGF-EGFR signaling^8^, although the process was not completely clarified. EGF mediates signaling via two major downstream mechanisms: the PI3K–Akt and Ras–Erk pathways^16^. Erk1/2 is transiently activated after a few minutes of glucose exposure in a concentration-dependent manner via Ca^2+^ entry and protein kinase A activation. Pharmacological blockade or depletion of Erk1/2 in MIN6 cells impairs insulin secretion^17^.

PLD2 is a Ca^2+^-dependent isozyme that is important for exocytosis, and it is also a downstream signaling effector for Erk^18^. PI3K–Akt signaling also plays a crucial function in the modulation of β-cell function; in particular, class IA PI3K regulates the secretion of the soluble N-ethylmaleimide attachment protein receptor (SNARE) proteins, which are responsible for insulin exocytosis^16^. Knockout of PI3K in mice causes glucose intolerance with decreased insulin secretion. Consistent with these findings, PRSS8 depletion suppressed EGFR downstream signaling and insulin secretion in vitro.

Because glucose induces plasma EGF elevation in vivo^8^, the contribution of EGFR signaling to GSIS appears reasonable. Interestingly, we found that EGFR was activated by short-term glucose exposure, and EGFR inhibition partially suppressed GSIS. Furthermore, short-term exposure to glucose increased EGF production in MIN6 cells. These observations indicate that EGF is secreted by β-cells themselves. In addition to exogenous ligands, EGFR is activated by autocrine, paracrine, and juxtacrine ligands^19^. The EGFR ligand, HB-EGF, is a known activator of its signaling^20^. In β-cells, glucose treatment for several days stimulates ChREBP and Src to improve HB-EGF gene release and HB-EGF shedding. Under this condition, no enhancement of insulin secretion was reported. This lack of enhancement may be due to long-term glucose exposure. Our results focusing on the short-term effects of glucose suggest that glucose induces EGF production by β-cells via insulin secretion.

Conversely, the propriety of EGFR signaling in pancreatic β-cells remains controversial. In patients with coincident lung cancer and type 2 diabetes mellitus, erlotinib improved fasting blood glucose levels and normalized HbA1c levels^21^. Li et al. reported that erlotinib treatment lowers blood glucose concentration and improved insulin tolerance and sensitivity^22^. They reported that EGFR blockade-induced insulin generation may occur due to a lower islet macrophage infiltration and higher islet autophagy in insulin-resistant model mice, manifesting with preserved islet structure and function. Because these studies focused on the long-term effects of EGFR signaling with limited applicability to pathological models, the short-term and physiological effects were fully investigated in the current study, thereby providing new insights into the effect of EGFR signaling and erlotinib in pancreatic β-cells.

Another major finding of our study was the regulation of PRSS8 by glucose. PRSS8 expression in islets was dynamically changed by diet, and this effect was likely dependent on the blood glucose concentration. In vitro, the glucose-dependent upregulation of PRSS8 expression was correlated with adequate insulin secretion. The finding of glucose-dependent PRSS8 regulation suggests the possibility of modulating EGFR activation in response to the demand for insulin secretion or proliferation. By contrast, long-term HSD feeding reduced PRSS8 expression in islets, suggesting its pathological contribution to diabetes. In vitro and ex-vivo experiments reproduced insulin secretion by short-term glucose loading, and insulin secretory function might be impaired by reduced pancreatic PRSS8 in obese conditions due to long-term HSD feeding. Our previous study revealed that hepatic PRSS8 expression was decreased by high-fat diet-induced endoplasmic reticulum stress^11^. Similarly, long-term metabolic stress may reduce PRSS8 expression in pancreatic β-cells.

Three notable limitations affected this study as follows. First, this study did not address all PRSS8 mechanisms in β-cells. On the basis of previous reports of PRSS8 activity, we initially assumed that it modulates insulin secretion by direct modification of ion channels; however, the expected results were not obtained (data not shown). PRSS8 might affect other substrates, such as PDGFR^12^. Second, we could not examine the effects of PRSS8 on insulin secretion and β-cell proliferation independently. In this study, a partial distinction was made using the time condition. Third, this study could not entirely investigate pathological modifications of PRSS8 in mouse pancreatic islets.

**Figure 7:**
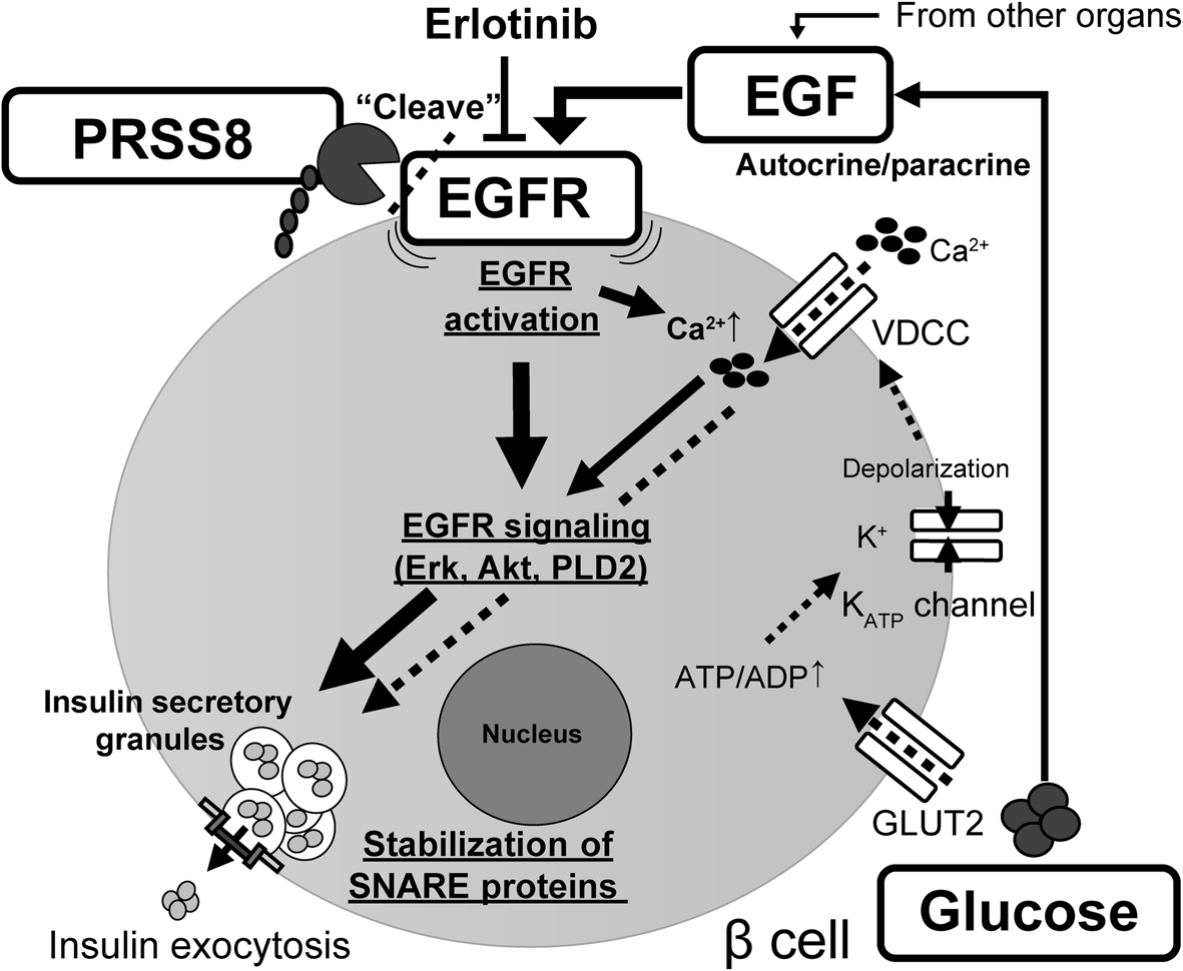
Proposed mechanism by which prostasin (PRSS8) controls β-cell insulin secretion via epidermal growth factor (EGF)–EGFR signaling. Glucose induces both the major pathway of insulin secretion and EGF production. EGF produced by β-cells and other cells activates EGFR. PRSS8 modulates EGFR activation according to its expression level. EGFR signaling triggers insulin exocytosis via Akt, Erk, phospholipase D2 (PLD2), and SNARE proteins. The use of erlotinib to block glucose-stimulated insulin secretion in this study is indicated. The solid line denotes the auxiliary pathway of insulin secretion via EGF–EGFR signaling. The dotted line denotes the major pathway of insulin secretion.

In conclusion, this study showed that PRSS8 is a potential regulator of insulin secretion through the EGF–EGFR signaling (Fig. 7). Because EGFR signaling has dual effects on insulin secretion and proliferation, further studies of PRSS8 will provide significant insights into the regulatory mechanisms of β-cell function. Our findings regarding the function of PRSS8 on insulin secretion may add to an improved understanding of the pathophysiologic mechanisms underlying the development of diabetes and indicate the potential usefulness of targeting PRSS8 in the treatment of diabetes.

## Research design and methods

### Animals

All animals were obtained, kept, taken care of, and utilized in accordance with the Guiding Principles in the Care and Use of Animals in the Field of Physiologic Sciences published by the Physiologic Society of Japan.

C57BL/6 mice were bought from CLEA Japan (Tokyo, Japan). The experimental outline for obtaining the PRSS8^lox^ and PRSS8^Δ^ alleles were as previously reported^11^. Rat insulin promoter-driven Cre transgenic (RIP-Cre) mice were bought from the Jackson Laboratory. Mice with a floxed PRSS8 allele (PRSS8^lox/lox^) were intercrossed with RIP-Cre^+/+^ mice to generate RIP-Cre^+/+^PRSS8^lox/lox^ mice. RIP-Cre^+/+^PRSS8^lox/+^ mice were crossed with PRSS8^lox/lox^ mice to obtain RIP-Cre^+/−^PRSS8^lox/lox^ (βKO) mice. βKO mice and RIP-Cre^+/−^ mice were used as controls. To obtain β-cell-specific PRSS8-overexpressing (βTG) mice, cDNA encoding human PRSS8 was cloned into the rat insulin II promoter expression vector. For transgenesis, purified construct DNA was microinjected into zygotes and transferred to pseudopregnant C57BL/6 J female mice. Transgene integration into the genome of founders was confirmed by Southern blotting. Three independent lines (6, 12, and 26 copies) were established, and the 26-copy line was used for this study. WT littermates were used as controls for βTG mice.

Mice were accommodated under a 12-h light–dark cycle and fed with a normal standard chow diet. All experiments were carried out with male littermates between 8 and 12 weeks of age, unless otherwise stated. To assess the fasting and refeeding conditions, mice were denied food for 16 h (fasting) and further fed for 16 h (refeeding), unless otherwise stated. All mice administered a NCD and water ad libitum, unless otherwise stated. Some mice were fed with an HSD (67 kcal%) once they were 10 weeks old. The IPGTT and ITT were performed using mice that were fasted overnight. For these tests, mice were given an intraperitoneal injection of glucose (1.5 gkg^−1^) and insulin (0.75 Ukg^−1^) and the blood glucose level was assayed immediately before and after the injection at varied times. The AUC of insulin secretion was calculated after subtraction of basal insulin levels. Pancreatic islets were isolated from mice as described previously^23^. In brief, after bile duct cannulation, digestion of the pancreas was performed via incubation with a mixture of 1.4 mg/mL type XI collagenase (Sigma-Aldrich) at 37 °C for 15 min. Thereafter, isolated islets were manually selected and maintained in RPMI-1640 medium supplemented with 10% (v/v) FBS, 0.1% (v/v) penicillin/streptomycin, and 20 mM L-glutamine at 37 °C and 5% CO_2_. Isolated islets were incubated in RPMI 1640 medium containing 11 mM glucose for 16 h and cultured in the presence of 3 mM glucose for 2 h before experiments.

### Cells

The MIN6 cell line was gifted from Junichi Miyazaki of Osaka University. MIN6 cells were kept in Dulbecco’s modified Eagle’s medium supplemented (DMEM) with 10% (v/v) fetal bovine serum (FBS), 0.1% (v/v) penicillin/streptomycin, and 50 µM β-mercaptoethanol at 37 °C and 5% CO_2_. HEK-293 cells were bought from ATCC and maintained in DMEM containing 10% FBS.

### Plasmids and transfection

For the gene-silencing experiments, platinum-E retroviral packaging cells (Cell Biolabs; San Diego, CA, USA) were transfected with mouse PRSS8 shRNA (Silencer Select shRNA, ID: S94451, Thermo Fisher Scientific) or control shRNA (ID: 4,390,844) using jetPRIME (Polyplus; New York, USA) as described by the manufacturers’ instructions. MIN6 cells were infected with the retroviral supernatant after 48 h and selected with 1 mg/mL puromycin for 1–2 weeks. Colonies were picked to generate monoclonal cell lines.

For the gene-overexpressing experiments, MIN6 cells were transfected with pcDNA3.1-PRSS8 (GenScript, ID: OHu16476), pcDNA3.1-mutant PRSS8 (alanine substitution at the active site), or pcDNA3.1-empty vector using jetPRIME.

For the transient transfection experiments, cells were harvested 48 h after transfection, and PRSS8 expression was determined by western blotting. Cells were selected with 25 mg/mL hygromycin using the same technique to generate stable cell lines.

### Reagents

Glucose, recombinant human EGF, and erlotinib were purchased from FUJIFILM Wako (Osaka, Japan). Recombinant human PRSS8 was created in our laboratory as described previously^11^. Cycloheximide (CHX) was purchased from Nacalai Tesque (Kyoto, Japan). MG132 was purchased from ChemScence (NJ, USA).

### Measurements of insulin and EGF secretion

MIN6 cells were seeded into a 24-well plate at 5.0 × 10^5^ cells/well and maintained for 2–3 days. After overnight serum starvation, cells were pre-incubated for 2 h in Krebs–Ringer bicarbonate containing HEPES (KRBH) buffer (120 mM NaCl, 4.7 mM KCl, 1.2 mM KH_2_PO_4_, 2.4 mM CaCl_2_, 1.2 mM MgCl_2_, 20 mM NaHCO_3_, and 10 mM HEPES) containing 3 mM glucose and 0.5% (v/v) bovine serum albumin (BSA) at pH 7.4. Cells were incubated in 3 or 20 mM glucose-containing KRBH buffer for 1 h, and the culture supernatant was recovered. Insulin and EGF levels were measured using a mouse insulin ELISA kit (FUJIFILM Wako Shibayagi) and a mouse EGF ELISA kit (Thermo Fisher Scientific), respectively, according to the manufacturers’ instructions.

### Quantitative real-time reverse transcriptase PCR

TaqMan probes for PRSS8, EGF, and 18S rRNA were purchased from Thermo Fisher Scientific. Total RNA was used for the real-time PCR, as described previously^11^.

### Western blotting

Tissue or cell lysates were prepared by homogenization in T-PER or M-PER (Thermo Fisher Scientific). Protein lysates were subjected to SDS–polyacrylamide gel electrophoresis and probed with the following primary antibodies: PRSS8 (BD Biosciences), EGF (Santa Cruz), EGFR (Cell Signaling Technology), p-EGFR (Tyr1068, Cell Signaling Technology), Akt-1 (Santa Cruz), p-Akt (Ser473, Cell Signaling Technology), Erk1/2 (p44/42 MAPK, Cell Signaling Technology), p-Erk1/2 (Cell Signaling Technology), β-actin (Cell Signaling Technology), and Na + /K + -ATPase α (Santa Cruz). For the detection of EGFR and p-EGFR, 3%–8% Tris–acetate gels (Thermo Fisher Scientific) were used, and for others, 4%–20% Tris–Glycine gels (Thermo Fisher Scientific) were used. At least three technical replicates were performed for all blots. Full-length blots are presented in the Supplementary files.

### Immunohistochemistry

Mice were deeply anesthetized and perfused through the heart with 4% paraformaldehyde (PFA) phosphate buffer (PB) solution (FUJIFILM Wako). The samples were fixed in 4% PFA and subsequently embedded in paraffin. Then, 3-mm sections were prepared and stained with hematoxylin and eosin, whereas other sections were processed for immunohistochemistry. Nonspecific binding of the antibodies was blocked with 3% BSA before incubation overnight at 4 °C with the primary antibodies. The following primary antibodies were used for immunostaining: PRSS8 (BD Biosciences and PA5-27,977, Thermo Fisher Scientific), EGFR (Cell Signaling Technology), p-EGFR (Cell Signaling Technology), and insulin (Dako). Secondary antibodies included Alexa-conjugated goat antimouse, antirabbit, and antiguinea pig immunoglobulin G (IgG) antibodies.

### Immunoelectron microscopy

The samples were perfused and fixed in 4% PFA and 0.1% glutaraldehyde in 0.1 M PB (pH 7.4) at 4 °C for 1 h, and were washed three times in 0.1-M PB for lasting for 15 min per cycle. The following protocol was carried out at Tokai Electron Microscopy (Nagoya, Japan). Graded ethanol solutions (50%, 70%) were used to dehydrate the samples at 4 °C lasting 30 min per cycle. The samples had a 50:50 mixture of ethanol and resin (LR white; London Resin Co. Ltd., Berkshire, UK) added to it three times lasting 30 min per cycle. After infiltration, the samples were incubated three times with 100% LR white at 4 °C for 30 min. The samples were moved into a fresh 100% resin and polymerized using an ultraviolet polymerizer at 4 C overnight. The polymerized resins were ultrathin-sectioned at 90 nm with a diamond knife using an ultramicrotome (Ultracut UCT; Leica, Vienna, Austria), and the sections were mounted on nickel grids. The grids were incubated with primary antibody (anti-PRSS8; PA5-27,977, Thermo Fisher Scientific) in 1% BSA in PBS at 4 °C overnight, and they were washed three times with 1% BSA in PBS for 1 min each time. They were subsequently incubated with the secondary antibody conjugated to 10-nm gold particles (goat antirabbit IgG pAb) for 2 h at room temperature. After washing with PBS, the grids were placed in 2% glutaraldehyde in 0.1 M PB. Then, the grids were dried and stained with 2% uranyl acetate for 15 min and lead stain solution (Sigma-Aldrich Co., Tokyo, Japan) at room temperature for 3 min. The grids were observed using a transmission electron microscope (JEM-1400Plus; JEOL Ltd., Tokyo, Japan) at an acceleration voltage of 100 kV. Digital images (3296 × 2472 pixels) were taken using a CCD camera (EM-14830RUBY2; JEOL Ltd., Tokyo, Japan).

### Double-layer fluorescent zymography

Membrane-fractionated cell extracts were electrophoresed by SDS-PAGE. After electrophoresis, SDS was completely replaced by incubating the gel in 2.5% Triton-X 100 for 30 min, and then incubating the gel twice in ultradiluted water for 5 min each time. The gel was equilibrated with reaction buffer (50 mM Tris–HCl, pH 8.3) at 37 °C for 30 min. Cellulose acetate membranes (SELECA®-V; Toyo Roshi Kaisha, Japan) pretreated with 10% glycerol at 37 °C for 30 min were permeabilized with Lys-His-Tyr-Arg-MCA, and a PRSS8-specific synthetic substrate was layered on the top of the gel. The gel and substrate were incubated at 37 °C for 2 h and irradiated with UV light.

### Statistical analyses

The significance of differences was assessed by the Kruskal–Wallis test or one-way ANOVA with Tukey’s post hoc test using SAS version 9.4. *p* value of < 0.05 was used to show statistical significance.

### Study approval

The study protocol was approved by the Animal Care Committee of Yamanashi University (A1-21, 2020-29).

### Approval for animal experiments

All animals used in this study were obtained, housed, cared for, and used in accordance with the Guiding Principles in the Care and Use of Animals in the Field of Physiologic Sciences published by the Physiologic Society of Japan, and the protocol was approved by the Animal Care Committee of Yamanashi University. The authors complied with the ARRIVE guidelines.

## Supplementary Information

Supplementary Information 1.

Supplementary Information 2.

## Data Availability

The datasets generated and/or analyzed during the present study are available from the corresponding author on reasonable request.
