# Supplementary material for: Membrane protease prostasin promotes insulin secretion by regulating the epidermal growth factor receptor pathway
Source: Sci Rep. 2023 Jun 5;13:9086. doi: 10.1038/s41598-023-36326-7 (PMC10241893; doi:10.1038/s41598-023-36326-7)
Supplement: Supplementary file 1 — Supplementary Information 1. [file 41598_2023_36326_MOESM1_ESM.pdf]

Full unedited gel for Figure 1e

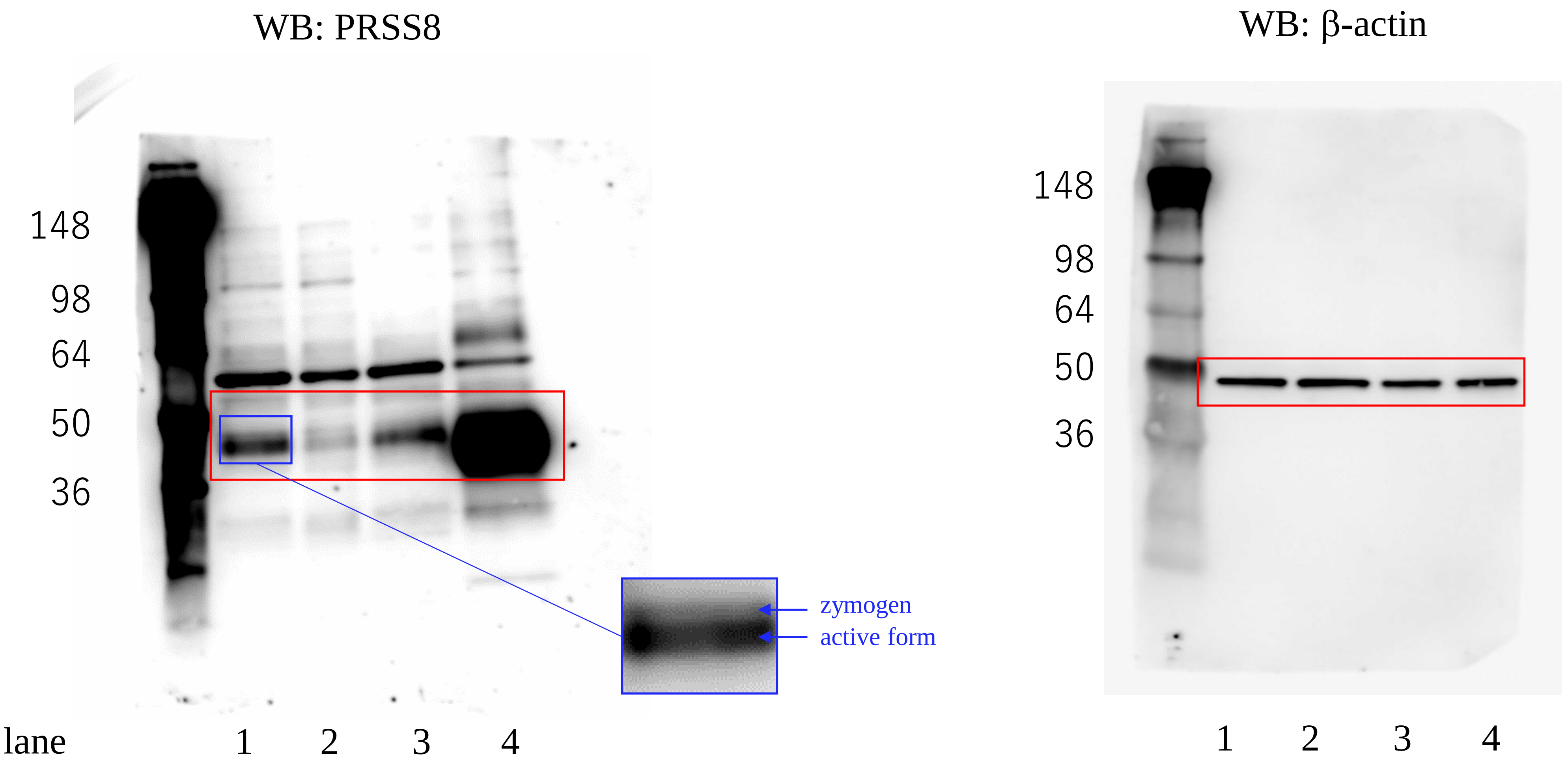

1: RIP-Cre
2: βKO
3: WT
4: βTG

Full unedited gel for Figure 3d

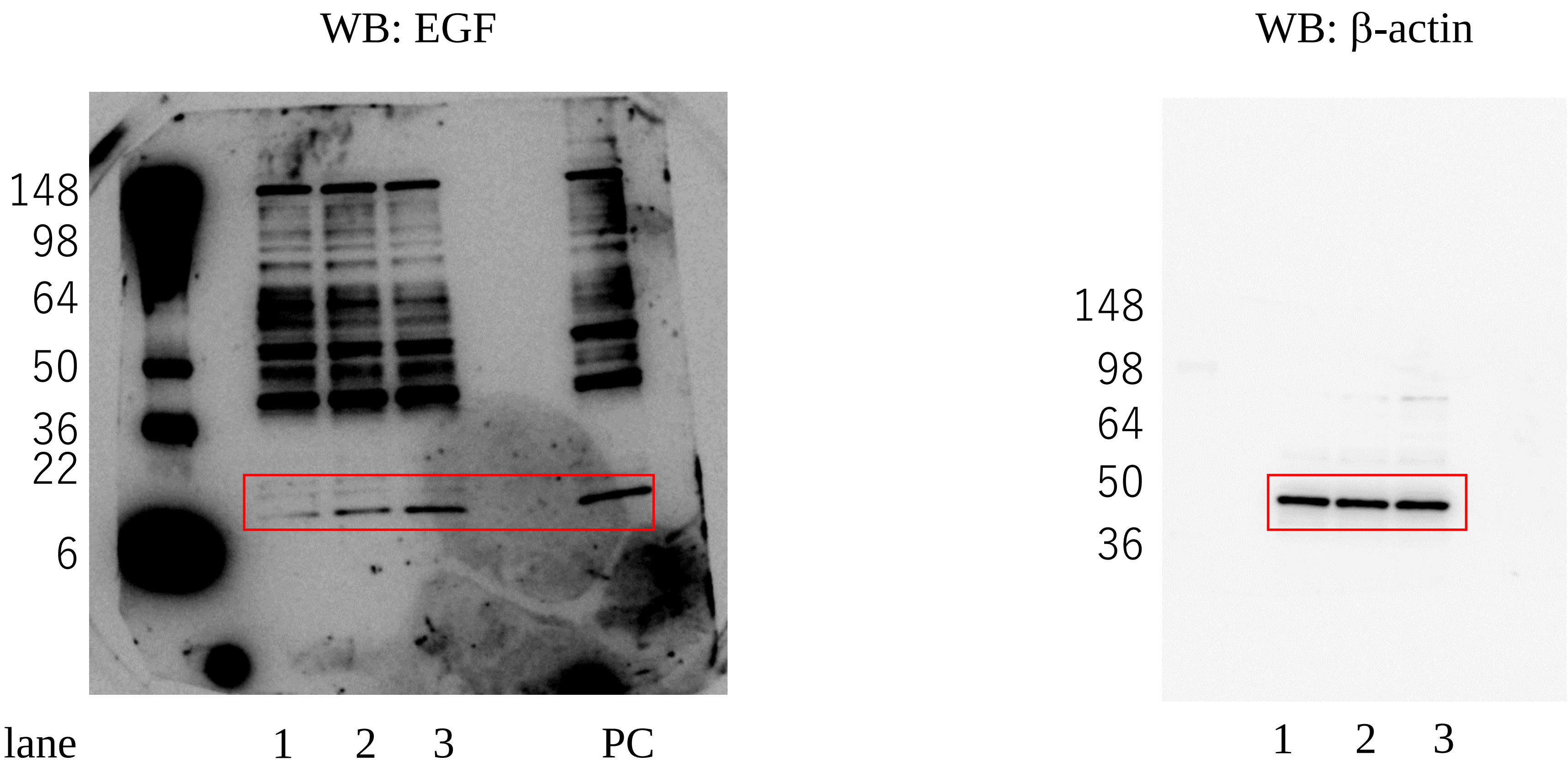

1 : Glucose(-), Erlotinib(-)
2 : Glucose(+), Erlotinib(-)
3 : Glucose(+), Erlotinib(+)
PC: recombinant human EGF+MIN6 lysate

Full unedited gel for Figure 4a

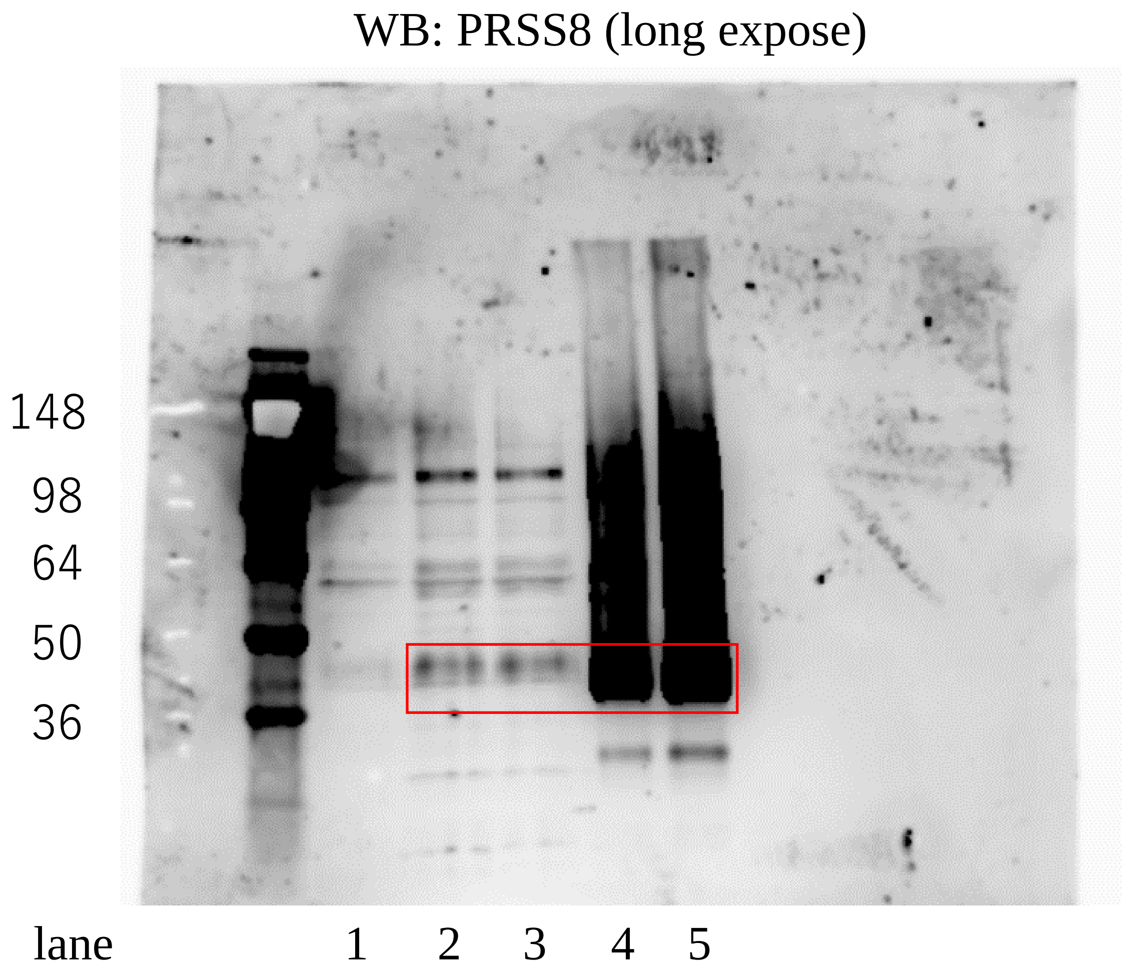

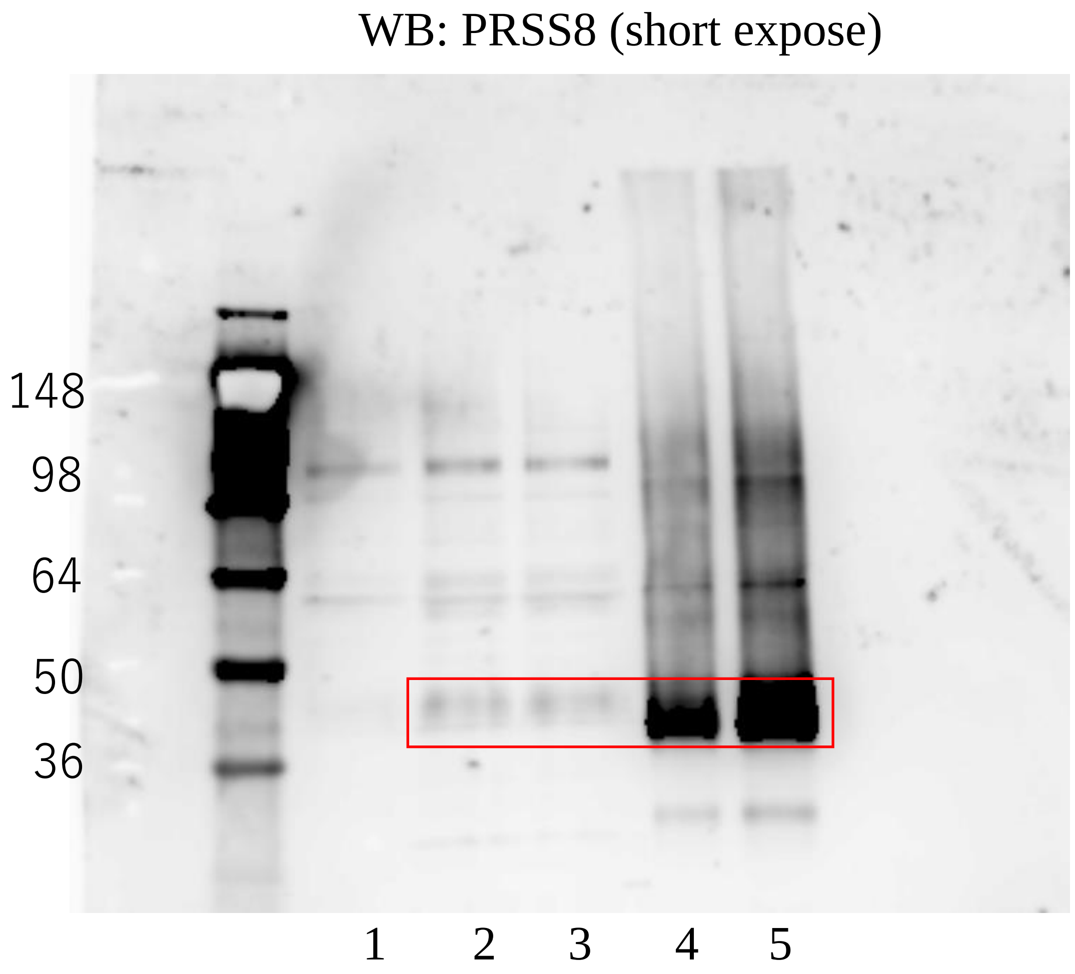

1: MIN6 (loading volume 10 μg)
2: MIN6 (loading volume 20 μg)
3: MIN6 (loading volume 30 μg)
4: Liver (positive control)
5: Liver (positive control)

Full unedited gel for Figure 4b

WB: PRSS8

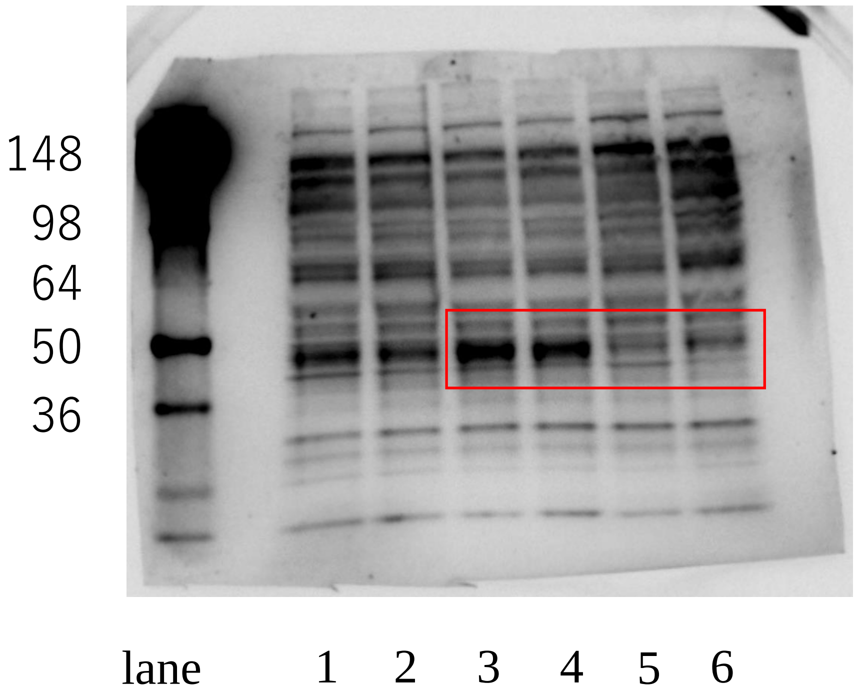

WB: β-actin

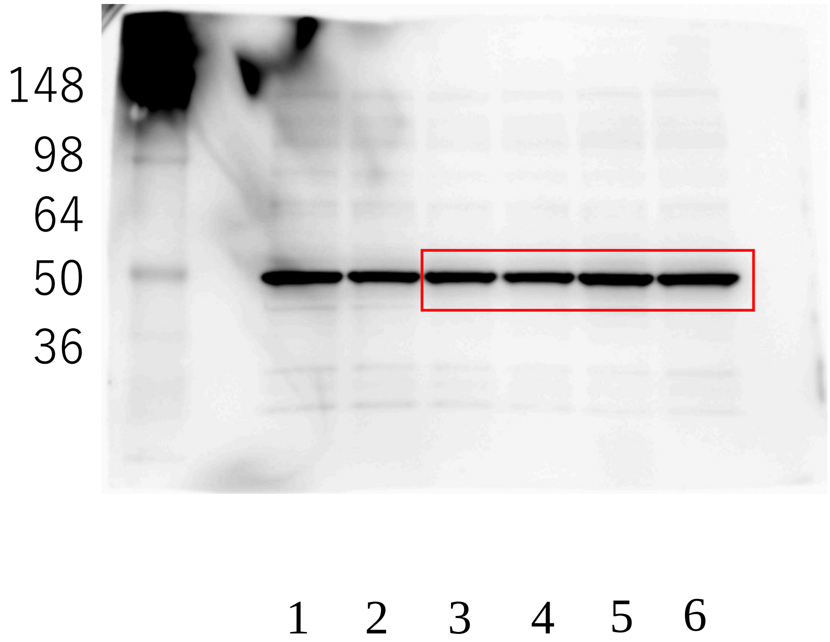

1: MIN6 (control)
2: MIN6 (control)
3: MIN6 (control)
4: MIN6 (control)
5: MIN6 (PRSS8 shRNA)
6: MIN6 (PRSS8 shRNA)

Full unedited gel for Figure 4d

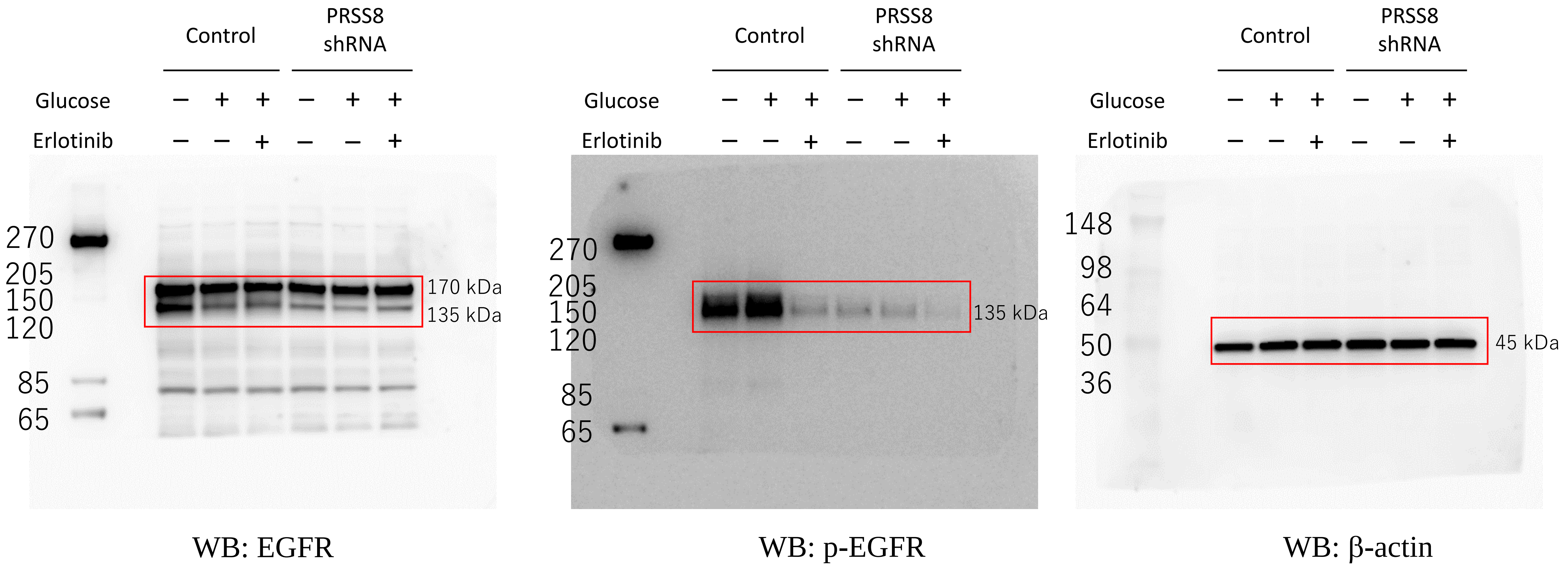

Full unedited gel for Figure 4e 1: control, Glucose (-) 2: control, Glucose (+) 3: shRNA, Glucose (-) 4: shRNA, glucose (+)

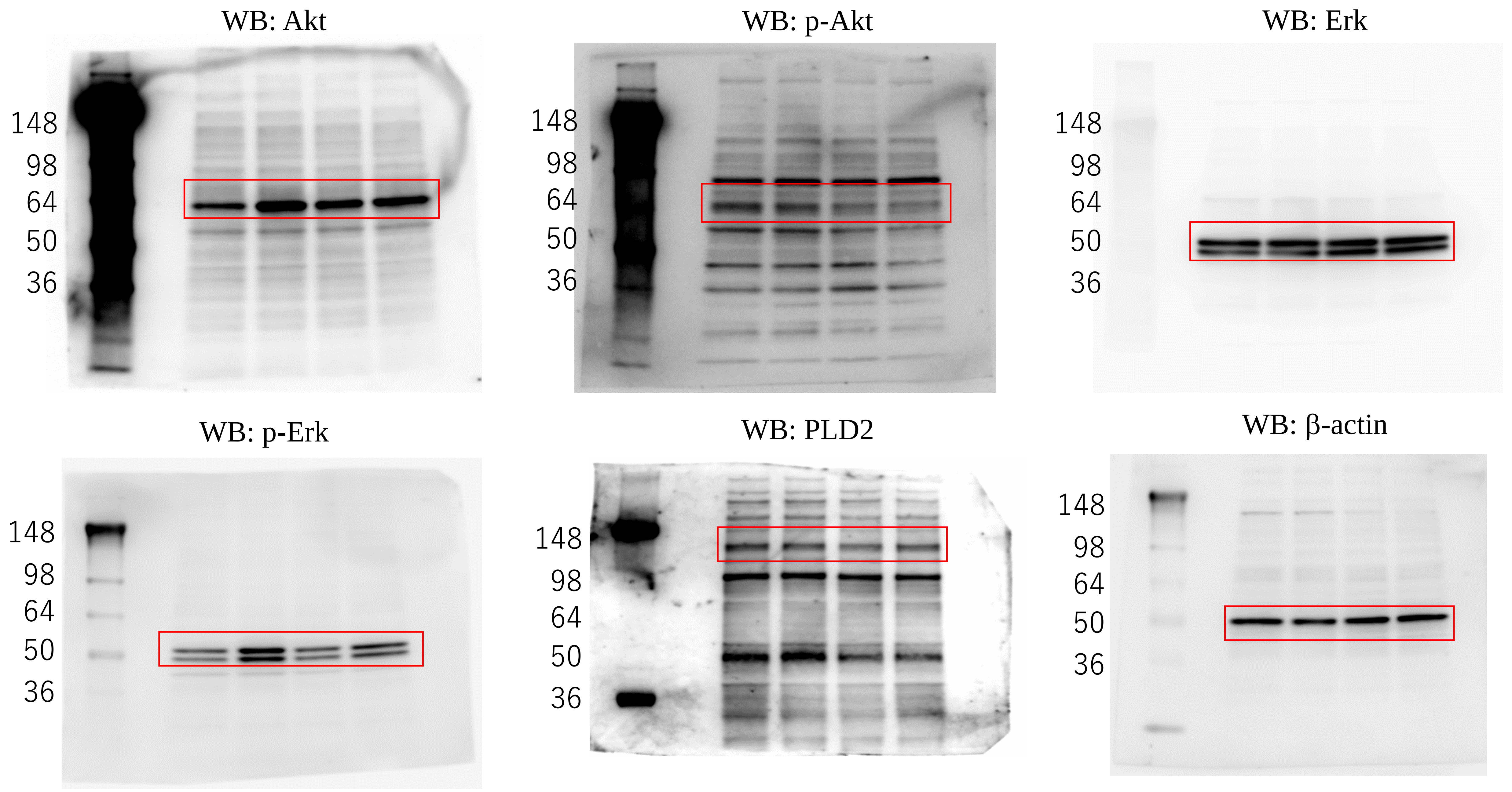

Full unedited gel for Figure 5a

WB: PRSS8

148
98
64
50
36

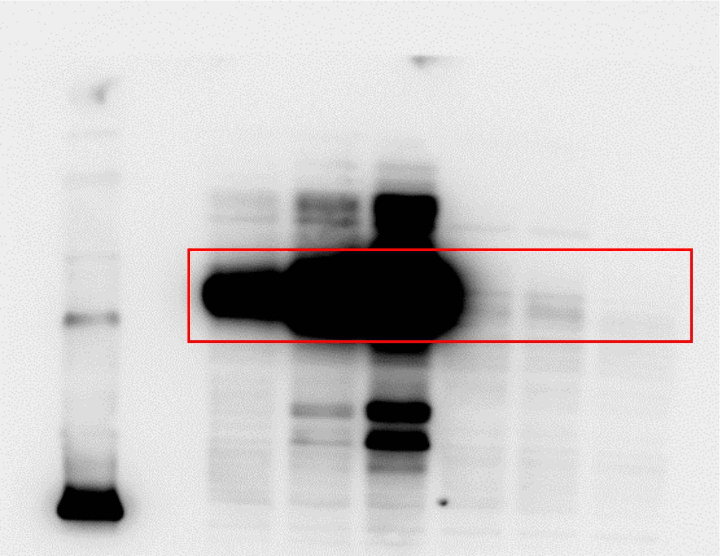

lane 4 5 6 7 8 9

WB: β-actin

148
98
64
50
36

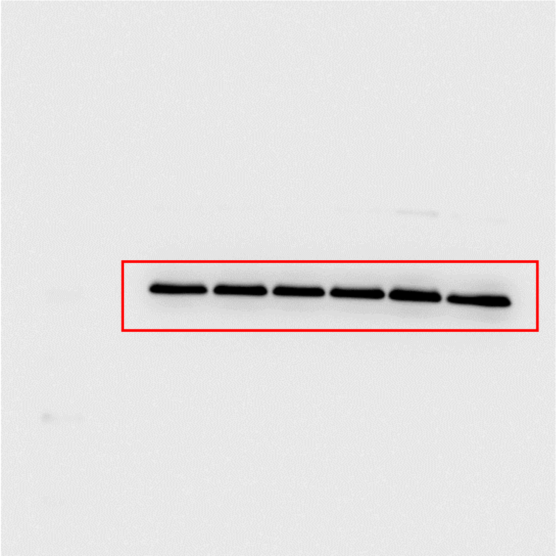

4 5 6 7 8 9

Activity (zymography)

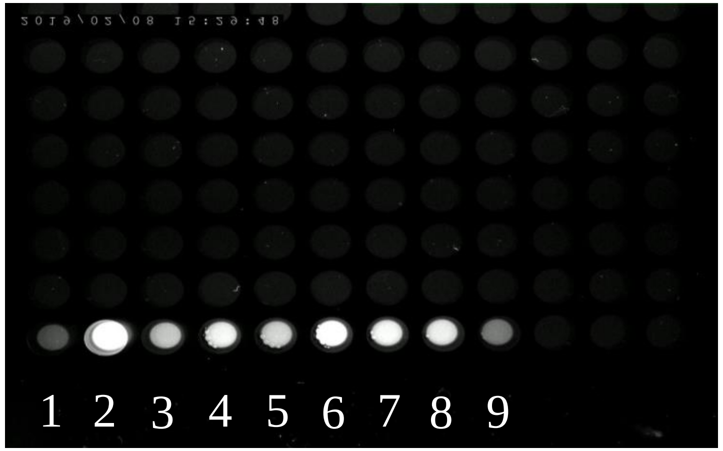

1: Negative control
2: Positive control (trypsin 1 mg)
3: recombinant human PRSS8
4: Intact #2
5: Intact #3
6: Intact #4
7: Empty #2
8: Empty #3
9: Empty #5

Full unedited gel for Figure 5c

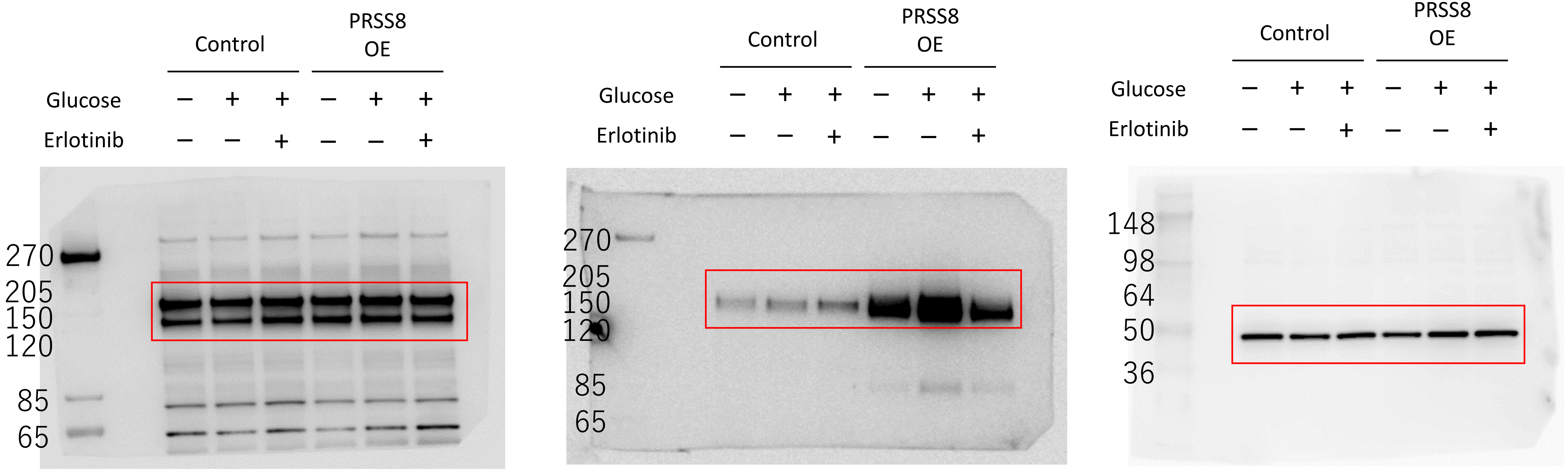

WB: EGFR WB: p-EGFR WB: β-actin

Full unedited gel for Figure 5d

Activity (zymography)

lane 1 2 3 4 5 6 7 8

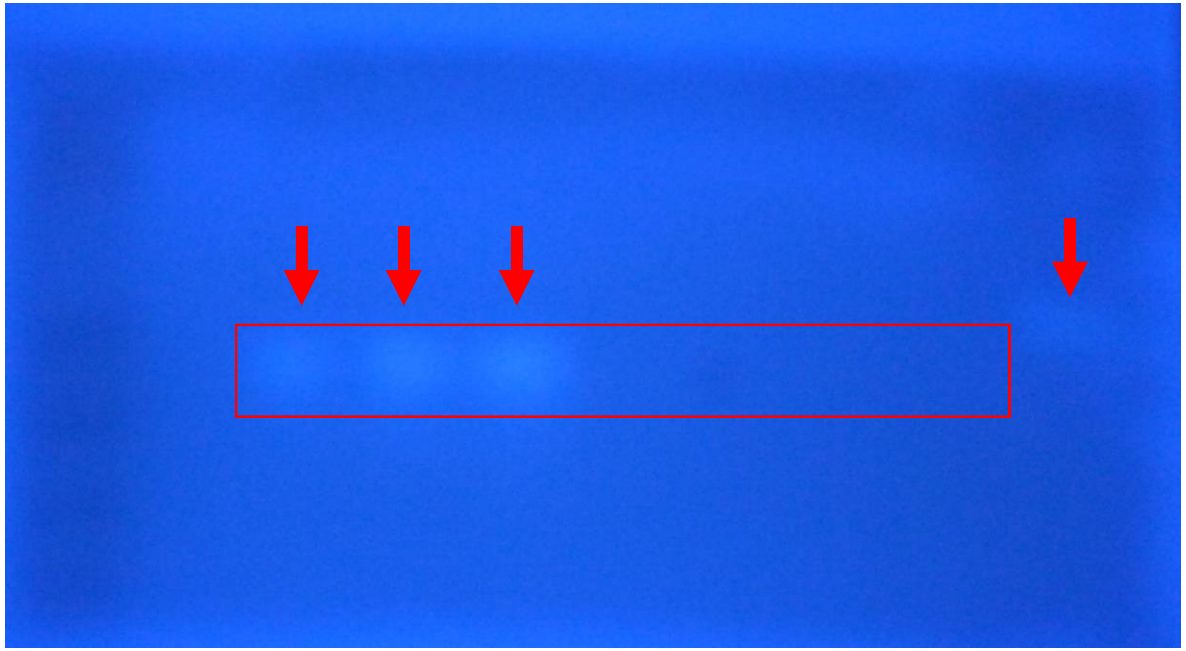

1: Intact (20 μg)
2: Intact (40 μg)
3: Intact (60 μg)
4: Blank
5: Mutant (20 μg)
6: Mutant (40 μg)
7: Mutant (60 μg)
8: Positive control (recombinant human PRSS8)

Full unedited gel for Figure 5e

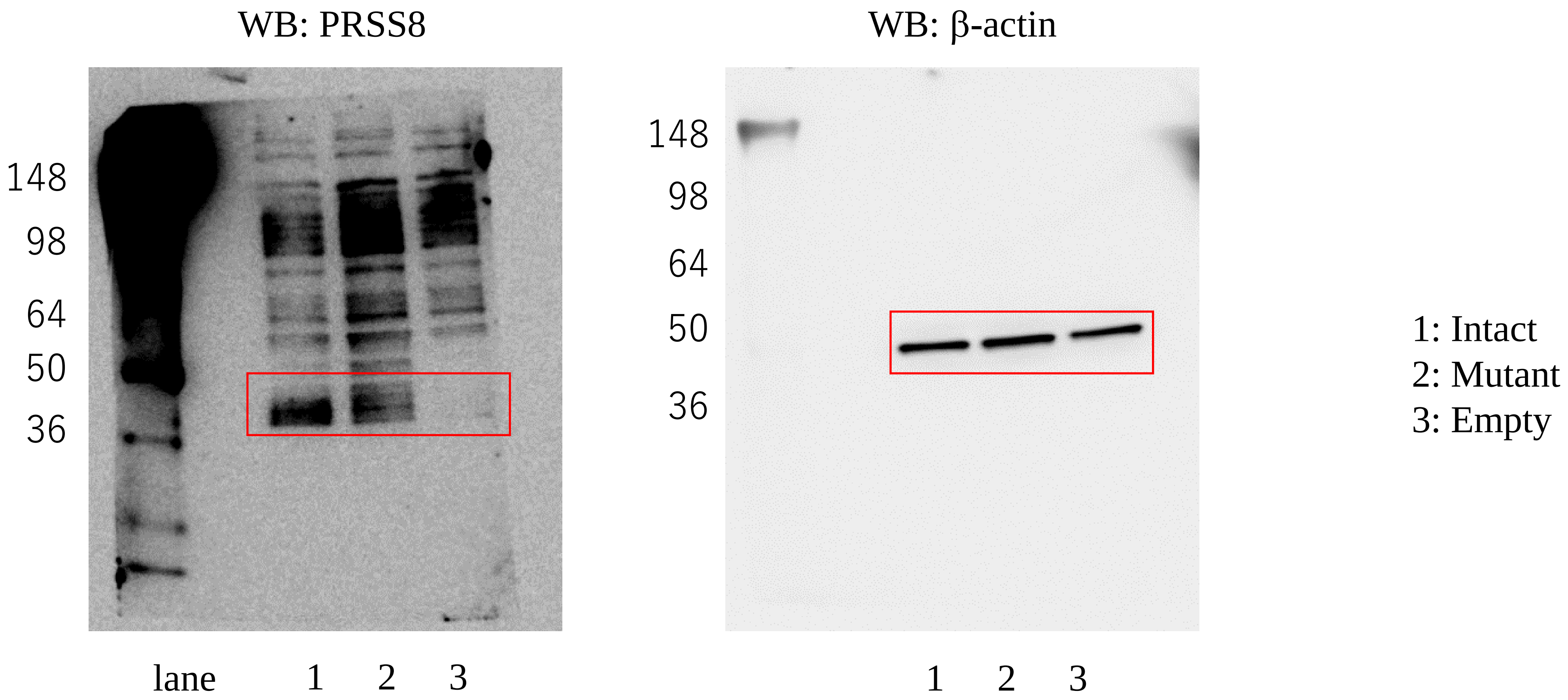

Full unedited gel for Figure 5h

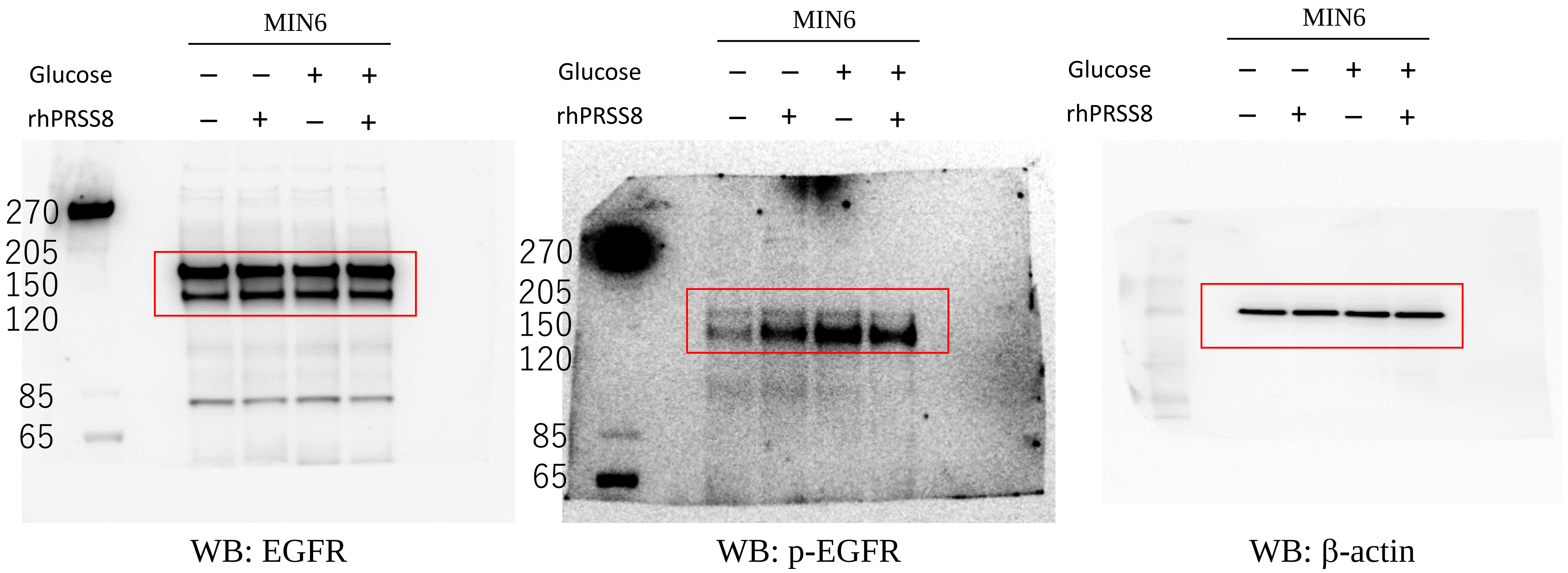

Full unedited gel for Figure 6a

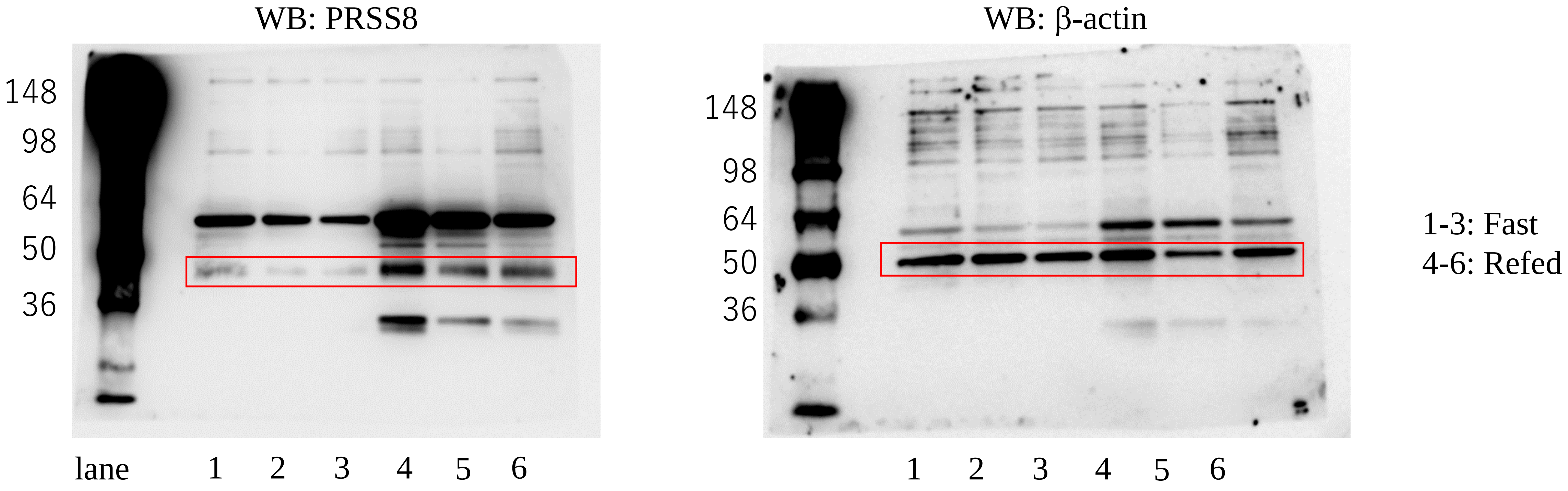

Full unedited gel for Figure 6b

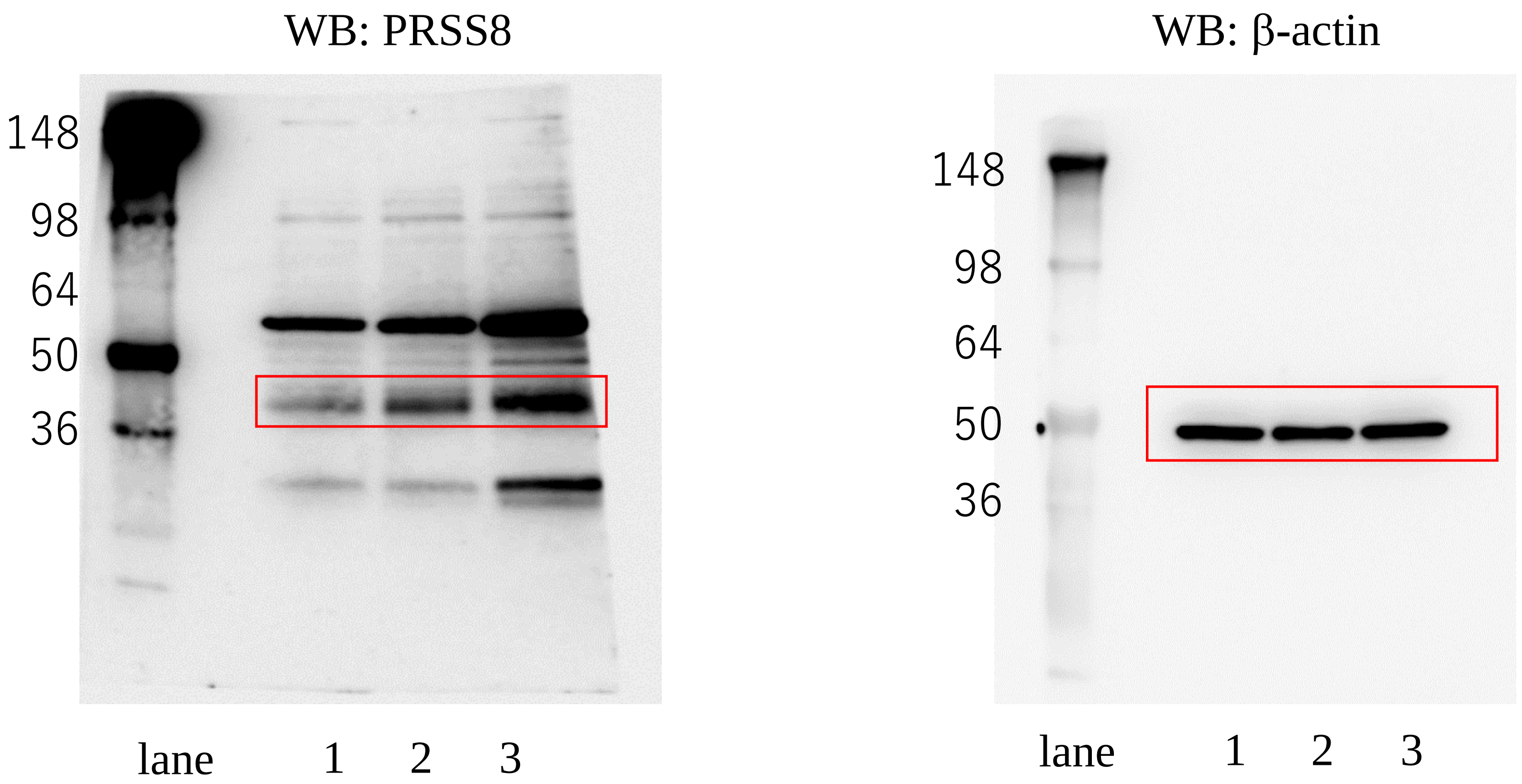

1: Fast (48h)
2: Fast (24h)
3: Refed

Full unedited gel for Figure 6c

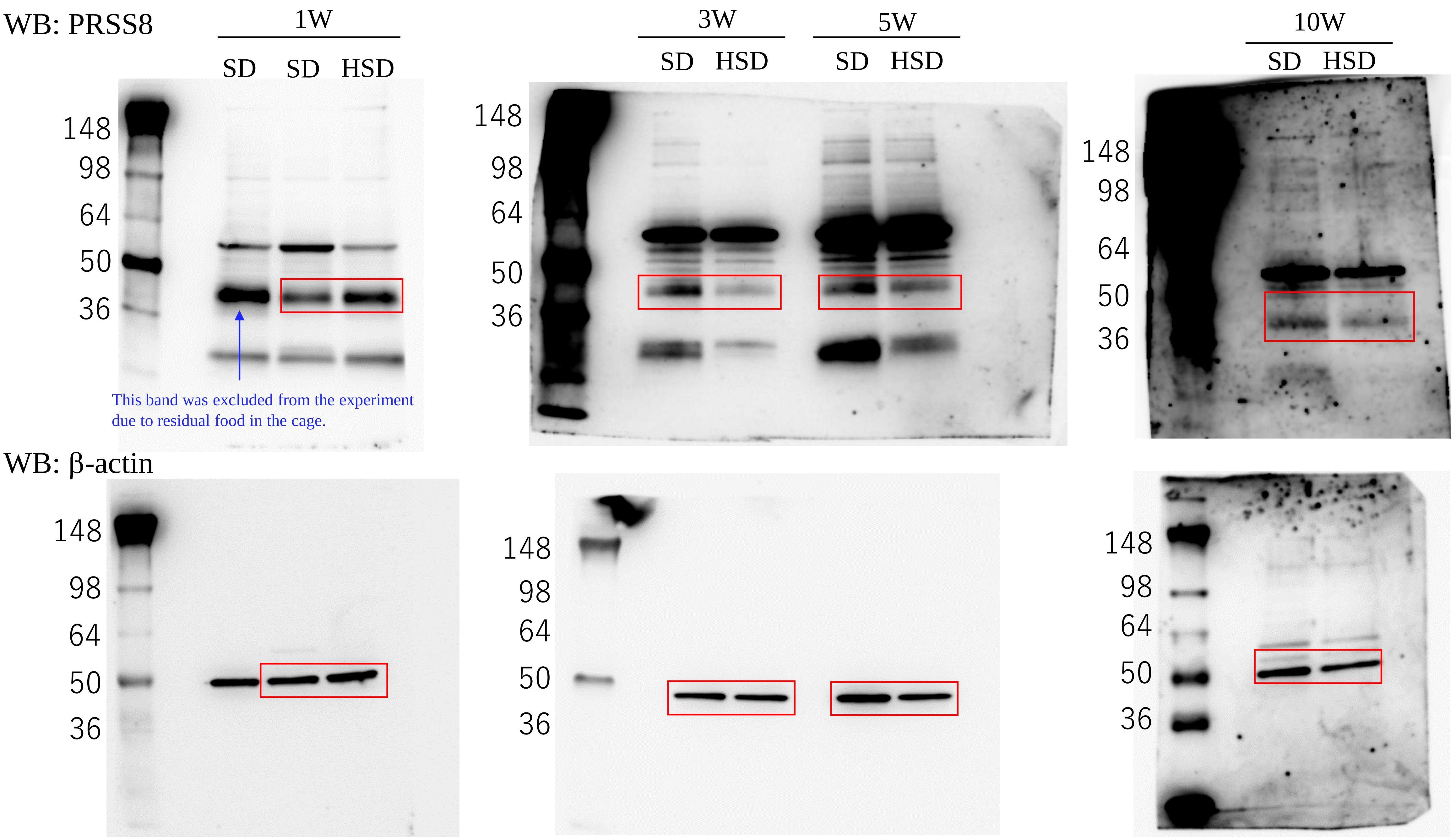

Full unedited gel for Figure 6d

WB: PRSS8

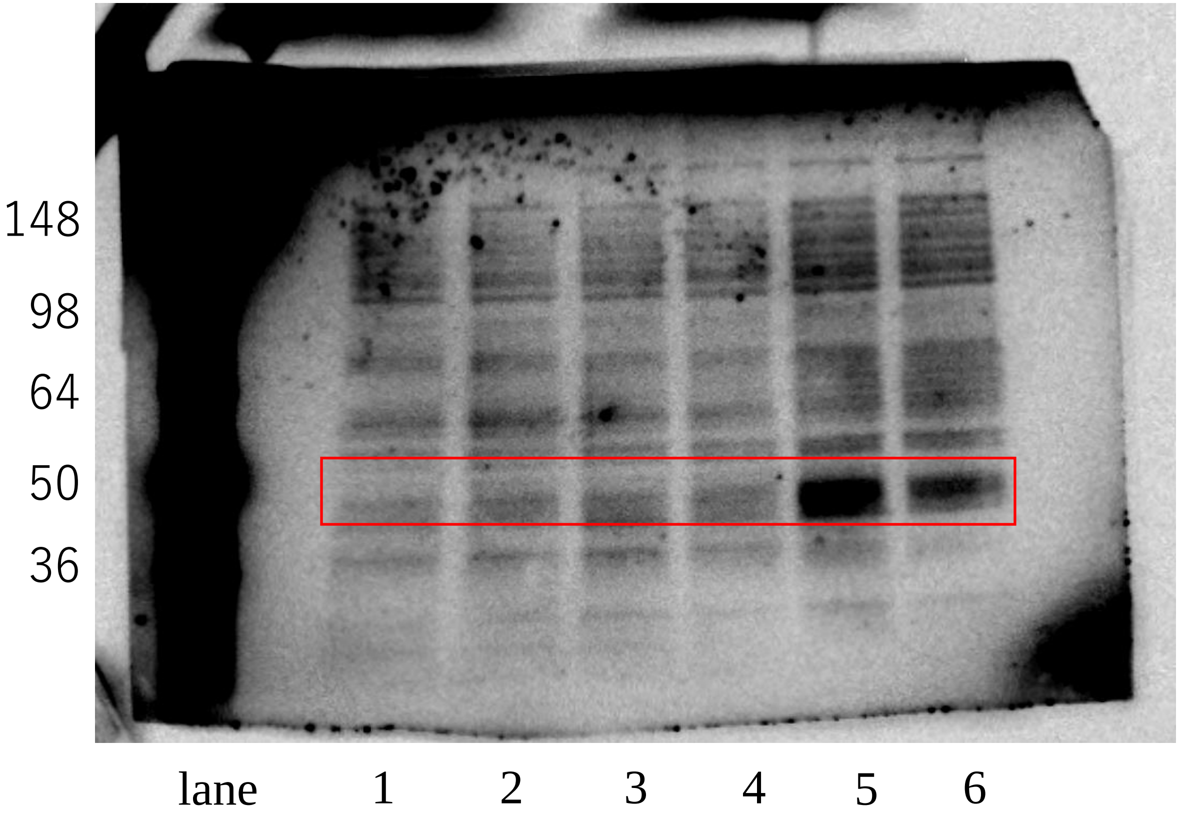

1-2: MIN6 (5 mMG)
3-4: MIN6 (10 mMG)
5-6: MIN6 (25mMG)

Full unedited gel for Figure 6f

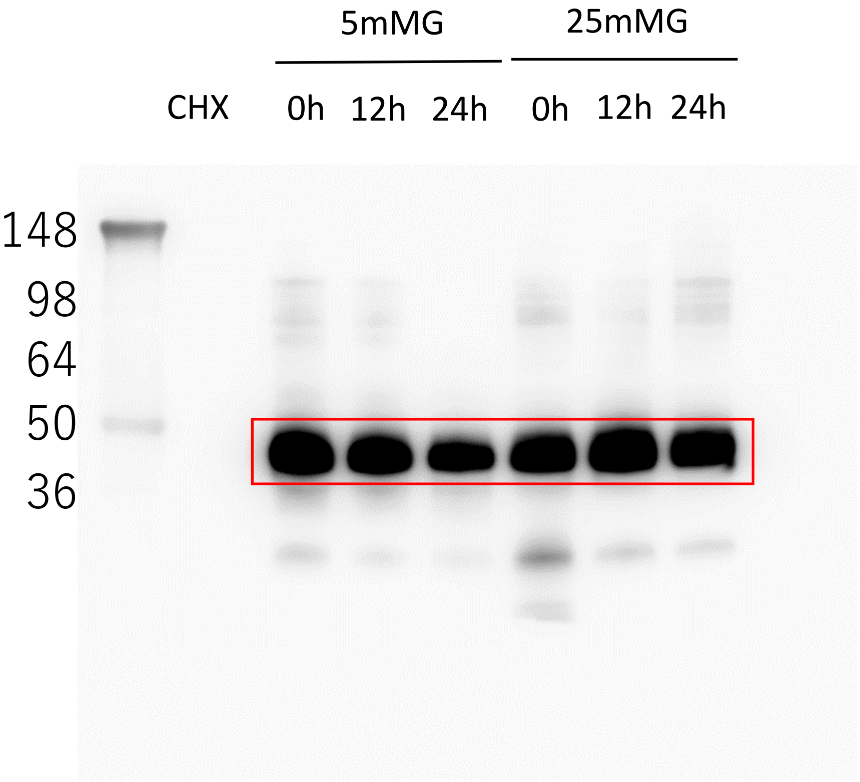

WB: PRSS8
(Membrane fraction)

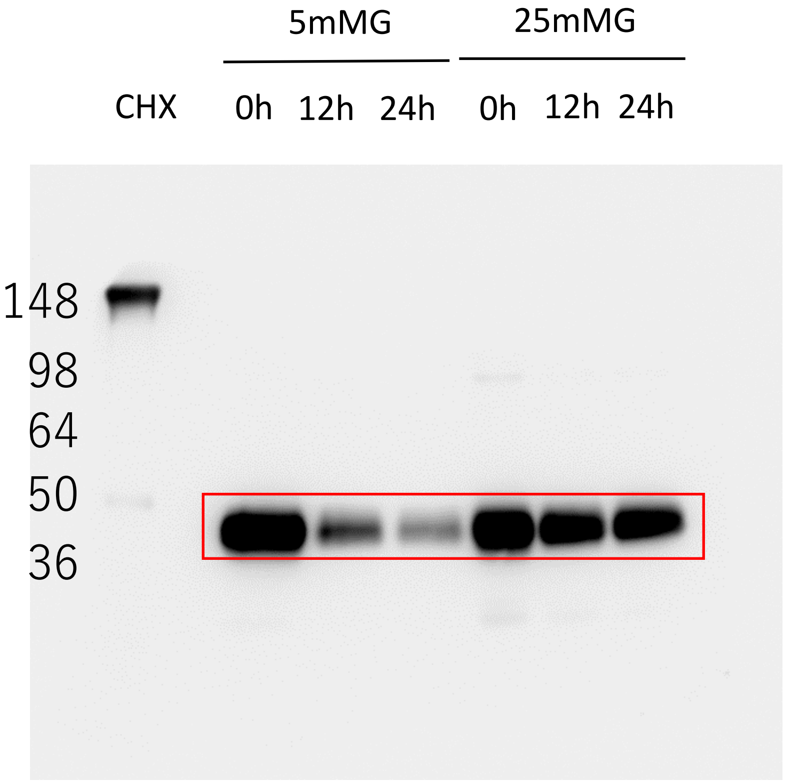

WB: PRSS8
(Whole cell lysate)

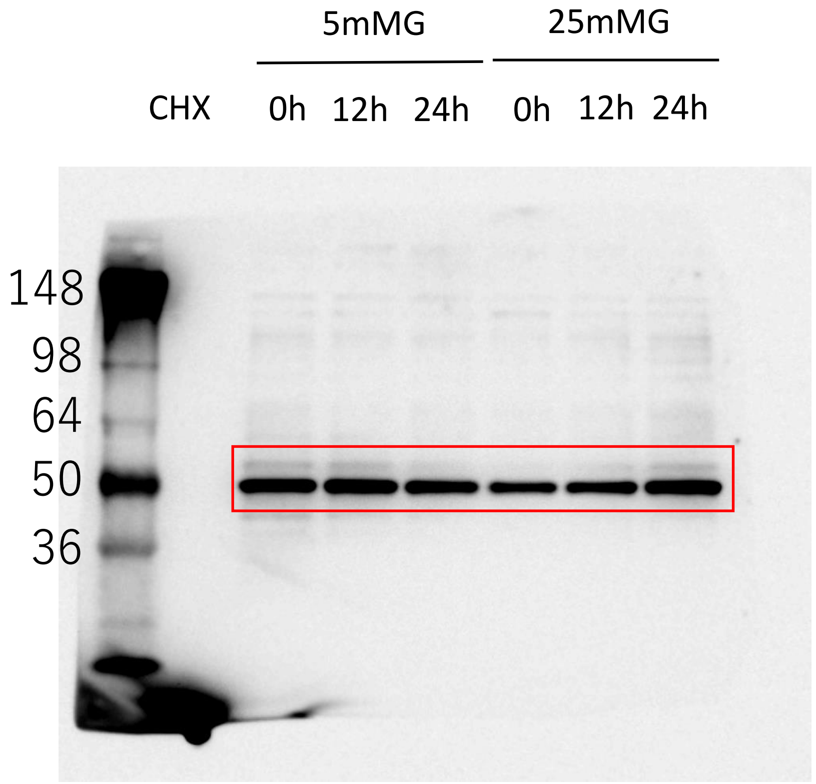

WB: β-actin

Full unedited gel for Figure 6g

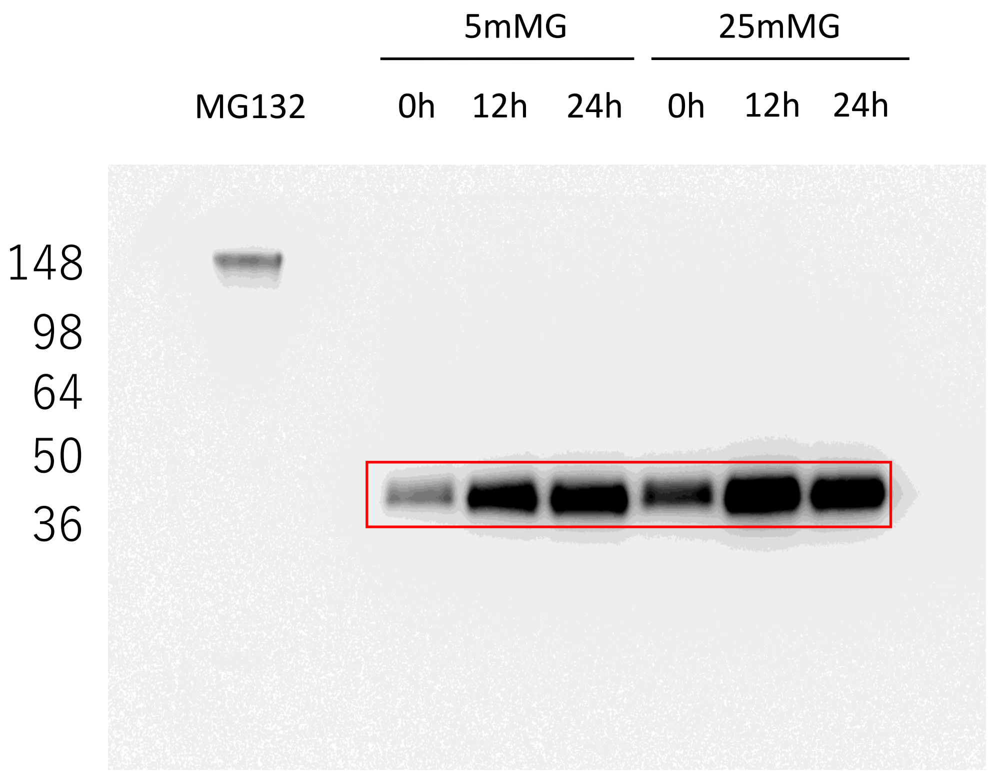

WB: PRSS8

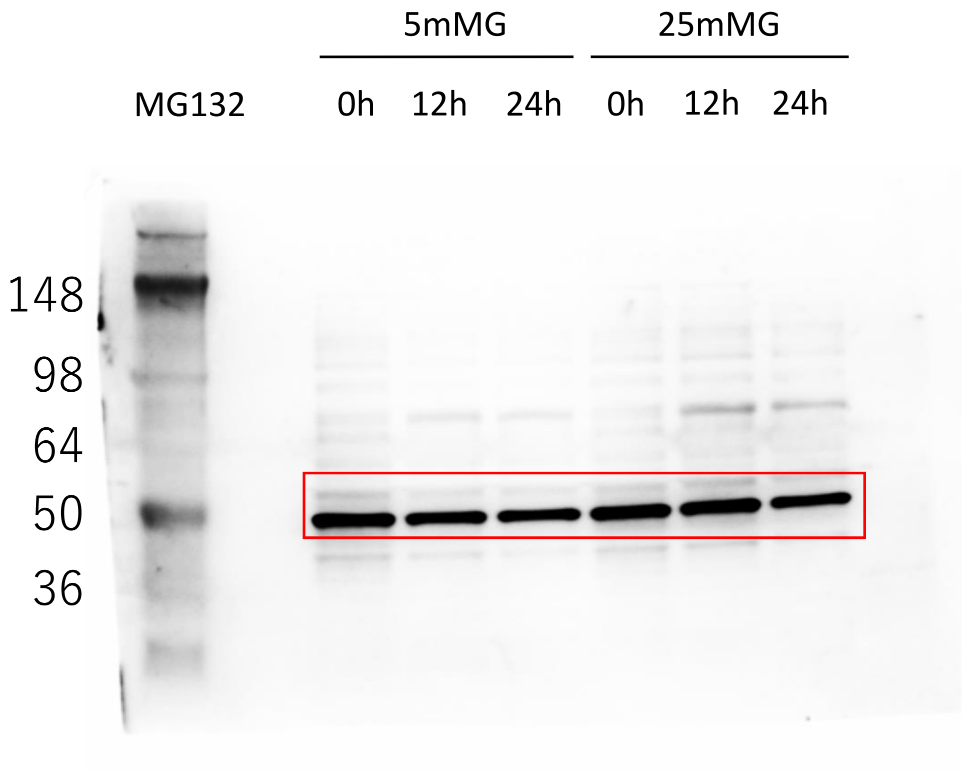

WB: β-actin
