# Supplementary material for: Membrane protease prostasin promotes insulin secretion by regulating the epidermal growth factor receptor pathway
Source: Sci Rep. 2023 Jun 5;13:9086. doi: 10.1038/s41598-023-36326-7 (PMC10241893; doi:10.1038/s41598-023-36326-7)
Supplement: Supplementary file 2 — Supplementary Information 2. [file 41598_2023_36326_MOESM2_ESM.pdf]

Supplementary Figure S1.

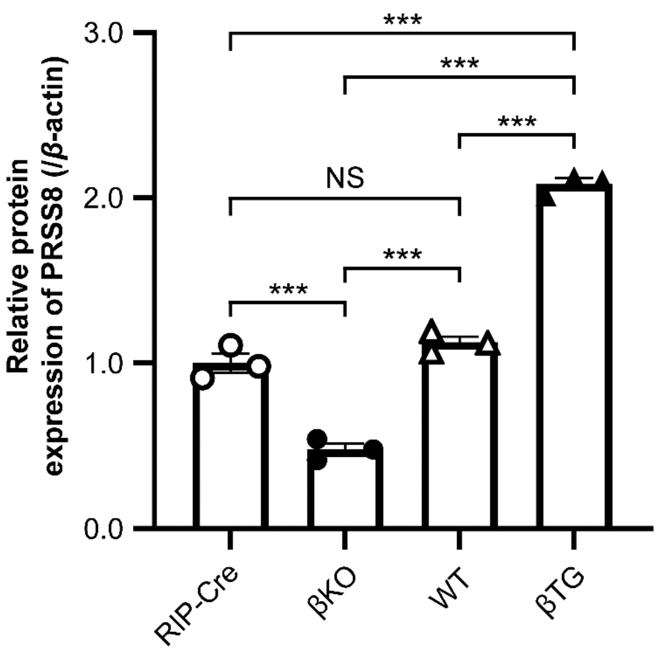


**Figure S1.** Relative protein levels for western blotting in Figure 1e (n=3/group). All data are presented as the mean ± SEM (error bars). NS, not significant; ***, $p < 0.001$.

Supplementary Figure S2.

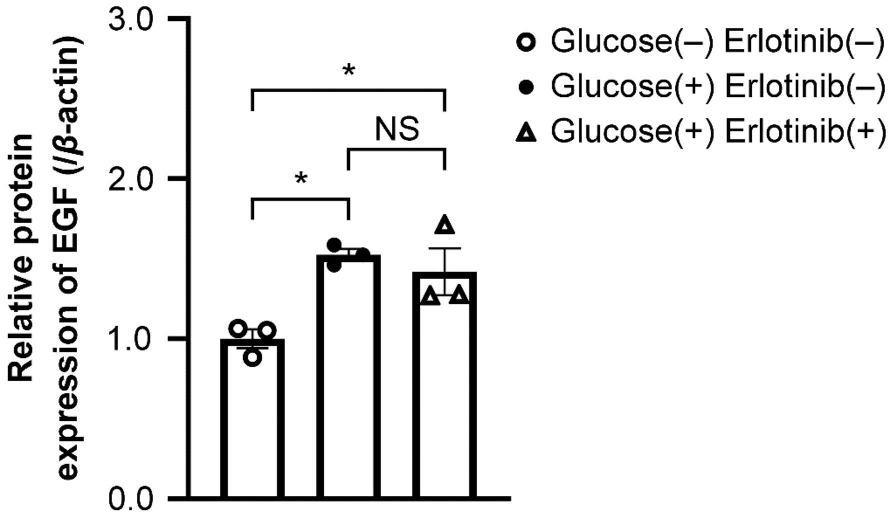


**Figure S2.** Relative protein levels for western blotting in Figure 3d (n=3/group). All data are presented as the mean ± SEM (error bars). NS, not significant; *, $p < 0.05$.

Supplementary Figure S3.

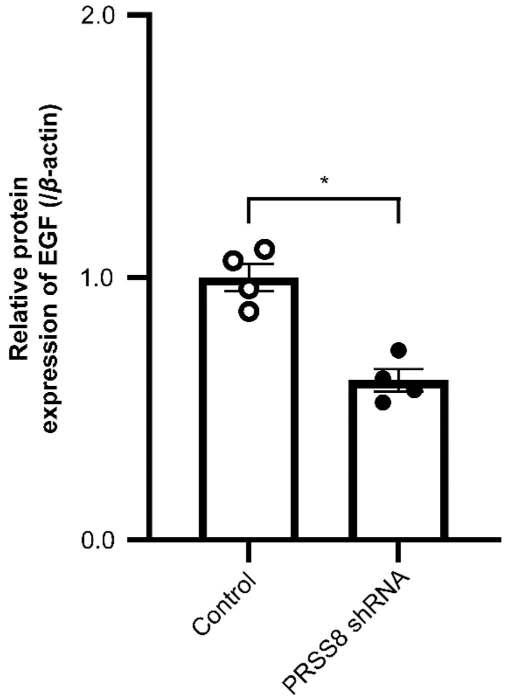


**Figure S3.** Relative protein levels for western blotting in Figure 4b (n=4/group). All data are presented as the mean ± SEM (error bars). *, $p < 0.05$.

Supplementary Figure S4.

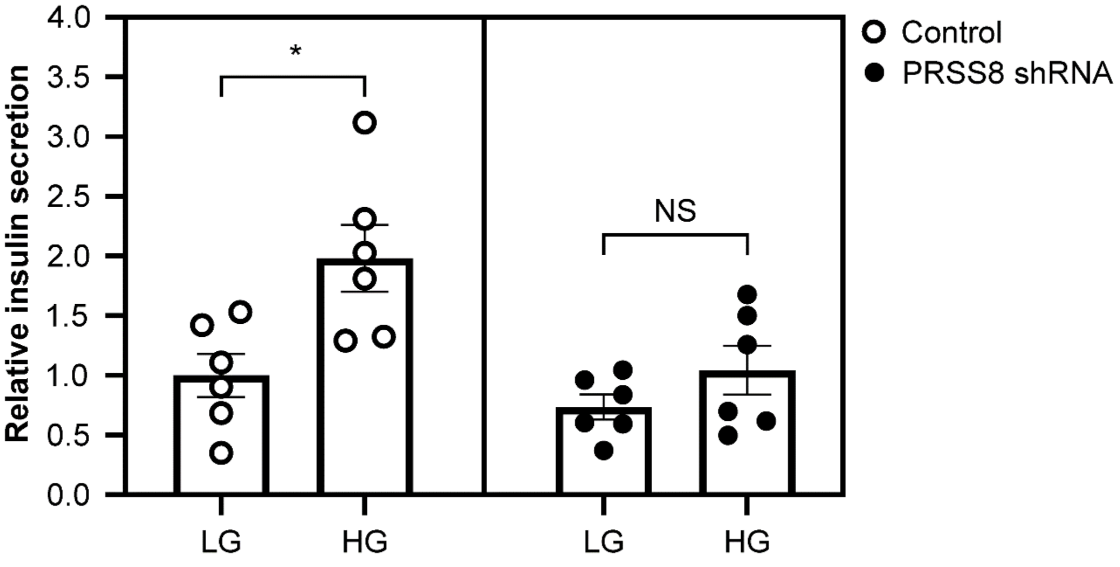


**Figure S4.** Pairwise comparisons supported in Figure 4c. GSIS in control and PRSS8-depleted MIN6 cells for 60 min (n = 6/group). Low glucose (LG), 3 mM glucose; high glucose (HG), 20 mM glucose. All data are presented as the mean ± SEM (error bars). NS, not significant; *, $p < 0.05$.

Supplementary Figure S5.

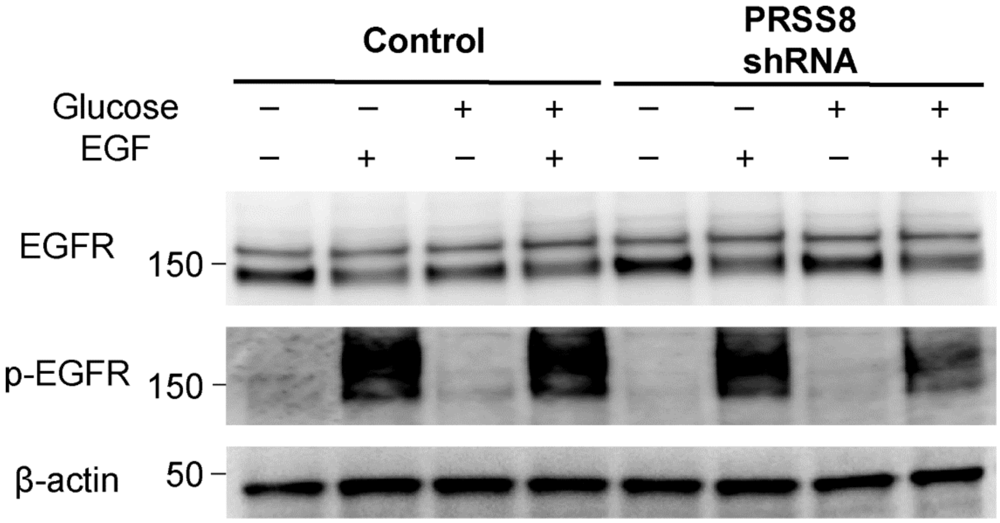


**Figure S5.** Western blotting for EGFR, p-EGFR, and β-actin in control vs. PRSS8-depleted cells treated with 3 mM, 20 mM glucose, and 50 nM epidermal growth factor (EGF). This experiment was repeated three times.

Supplementary Figure S6.

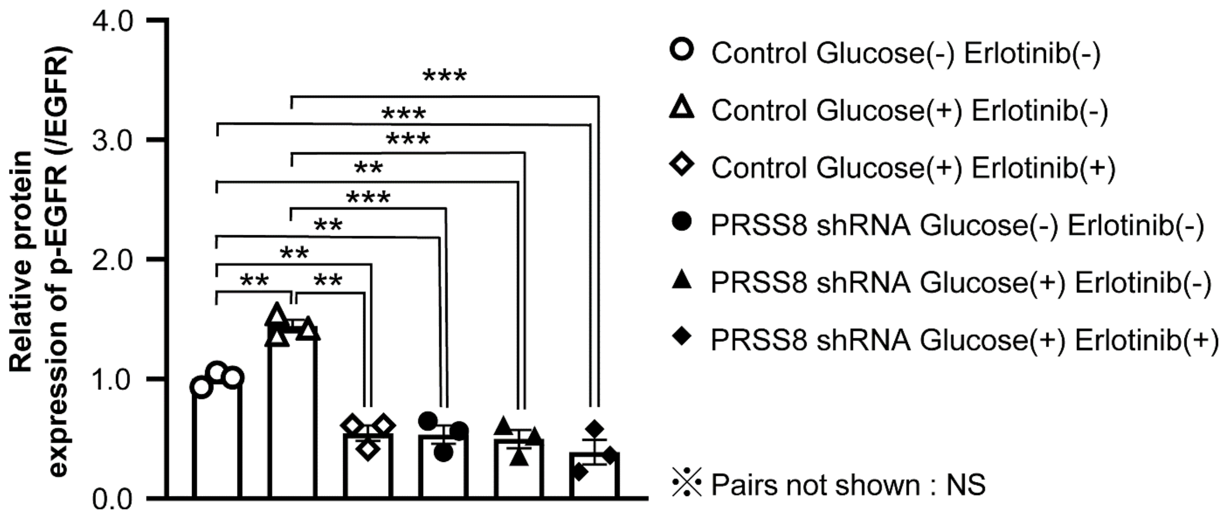


**Figure S6.** Relative protein levels for western blotting in Figure 4d (n=3/group).All data are presented as the mean ± SEM (error bars). NS, not significant; **, $p < 0.01$; ***, $p < 0.001$.

Supplementary Figure S7.

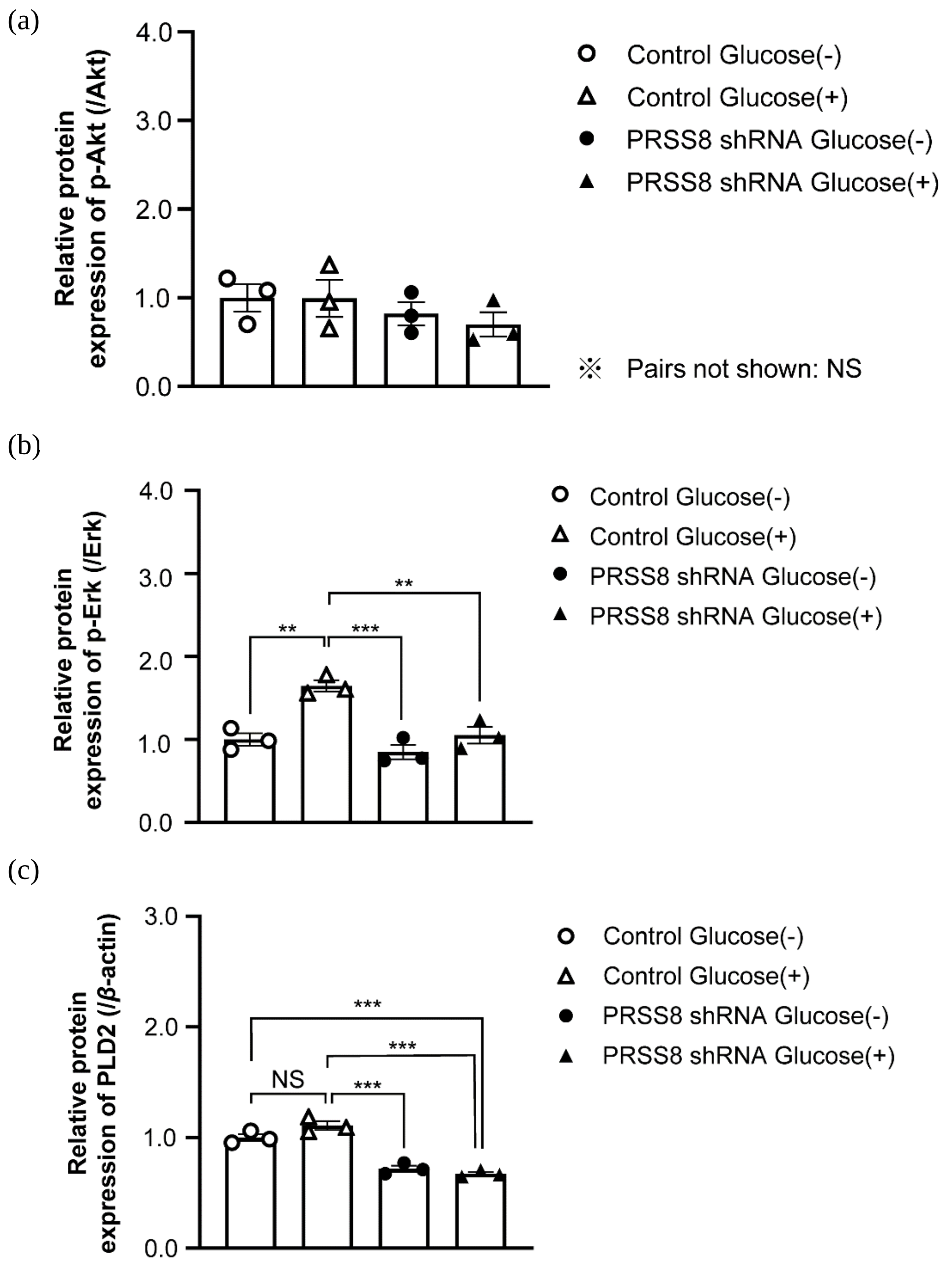


**Figure S7.** Relative protein levels for western blotting in Figure 4e (n=3/group). (a) p-Akt. (b) p-Erk. (c) PLD2. All data are presented as the mean ± SEM (error bars). NS, not significant; **, $p < 0.01$; ***, $p < 0.001$.

Supplementary Figure S8.

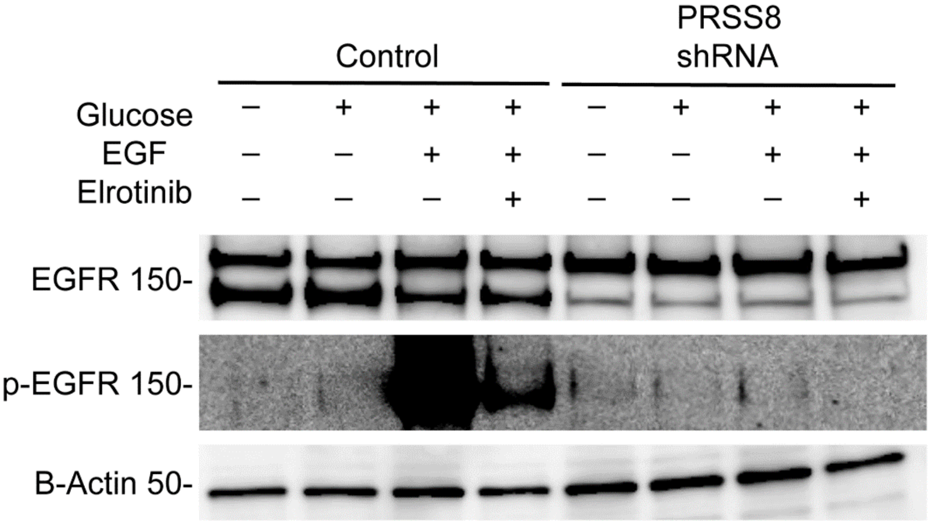


**Figure S8.** Western blotting for EGFR, p-EGFR, and β-actin in control vs. PRSS8-depleted cells (PRSS8 shRNA) treated with 3 mM, 20 mM glucose, 50 nM epidermal growth factor (EGF), and 30 nM erlotinib. This experiment was repeated three times.

Supplementary Figure S9.

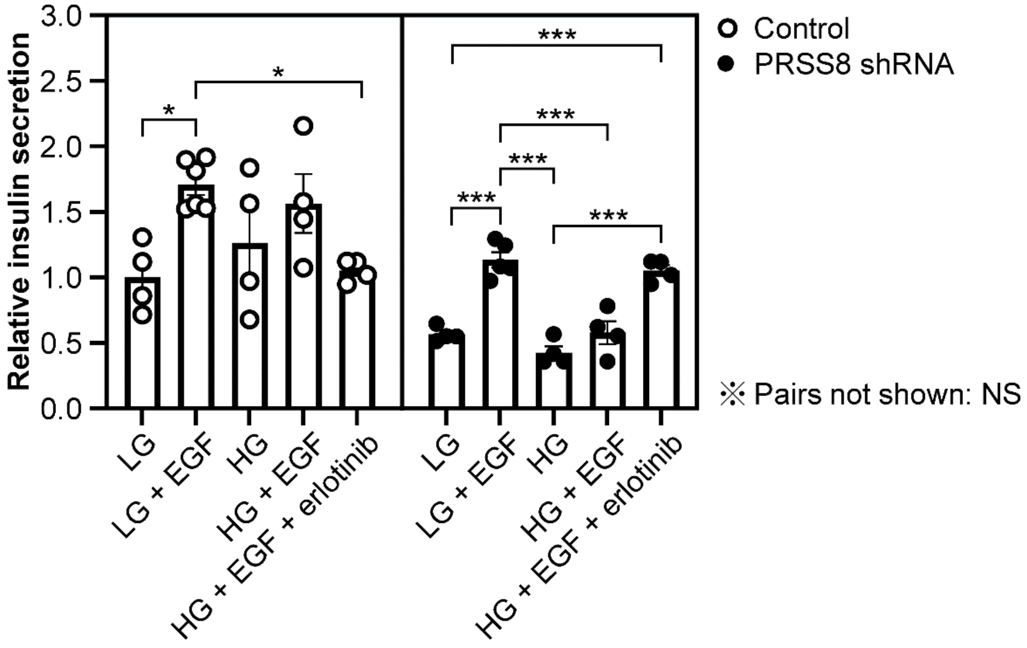


**Figure S9.** Pairwise comparisons supported in Figure 4f. Insulin secretion in PRSS8-depleted cells following treatment with 50 nM epidermal growth factor (EGF) and 30 nM erlotinib (n = 4/group). LG, 3 mM glucose; HG, 20 mM glucose. All data are presented as the mean ± SEM (error bars). NS, not significant; *, $p < 0.05$; ***, $p < 0.001$.

Supplementary Figure S10.

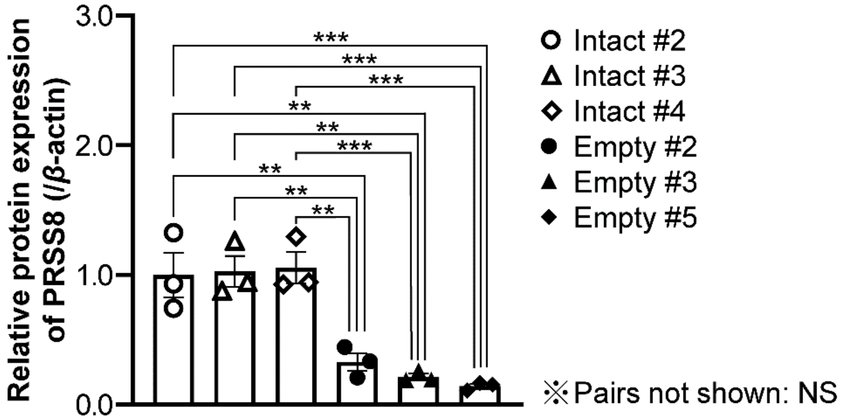


**Figure S10.** Relative protein levels for western blotting in Figure 5a (n=3/group). All data are presented as the mean ± SEM (error bars). NS, not significant; **, $p < 0.01$; ***, $p < 0.001$.

Supplementary Figure S11.

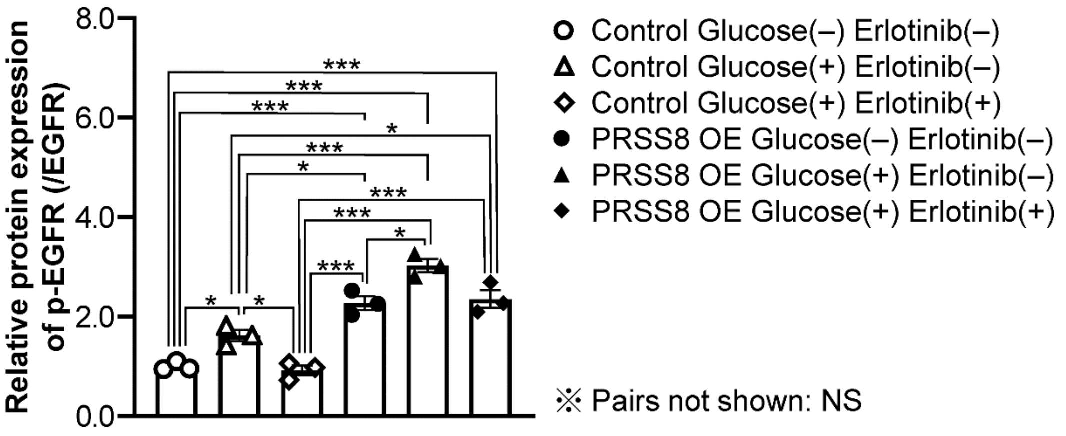


**Figure S11.** Relative protein levels for western blotting in Figure 5c (n=3/group). All data are presented as the mean ± SEM (error bars). NS, not significant; *, p < 0.05; ***, p < 0.001.

Supplementary Figure S12.

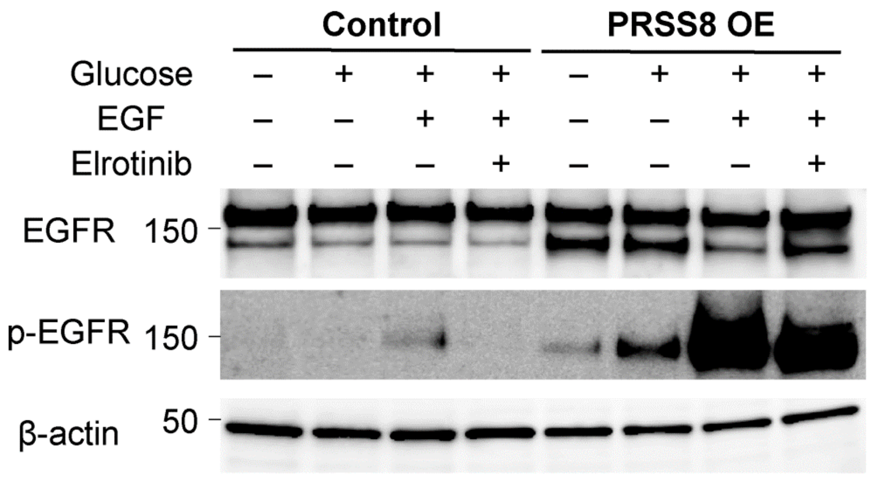


**Figure S12.** Western blotting for EGFR, p-EGFR, and β-actin in control vs. PRSS8-overexpressing (OE) cells treated with 3 mM, 20 mM glucose, 50 nM epidermal growth factor (EGF), and 30 nM erlotinib. This experiment was repeated three times.

Supplementary Figure S13.

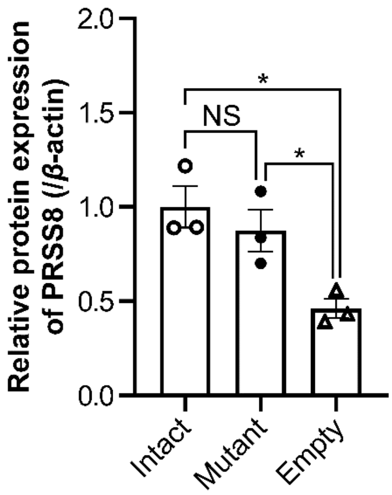


**Figure S13.** Relative protein levels for western blotting in Figure 5e (n=3/group). All data are presented as the mean ± SEM (error bars). NS, not significant; *, $p < 0.05$.

Supplementary Figure S14.

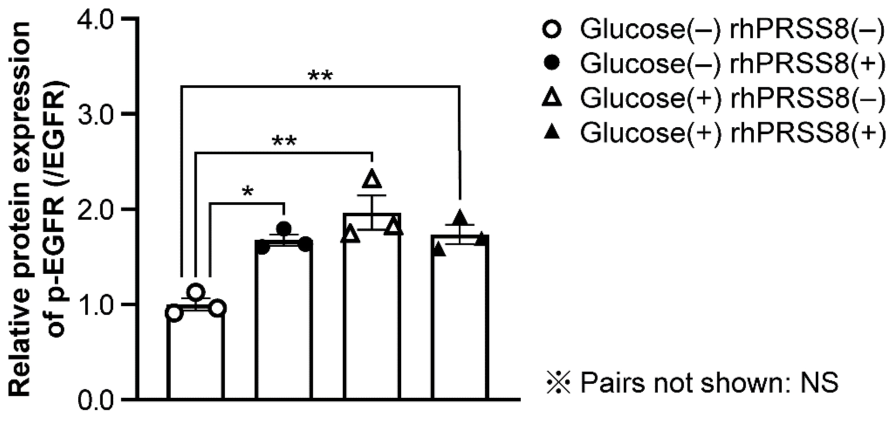


**Figure S14.** Relative protein levels for western blotting in Figure 5h (n=3/group). All data are presented as the mean ± SEM (error bars). NS, not significant; *, $p < 0.05$; **, $p < 0.01$.

Supplementary Figure S15.

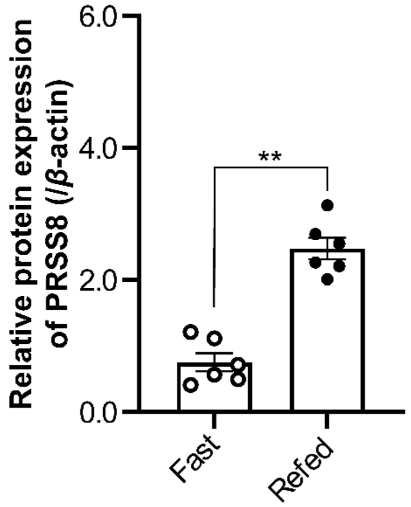


**Figure S15.** Relative protein levels for western blotting in Figure 6a (n=6/group). All data are presented as the mean ± SEM (error bars). **, $p < 0.01$.

Supplementary Figure S16.

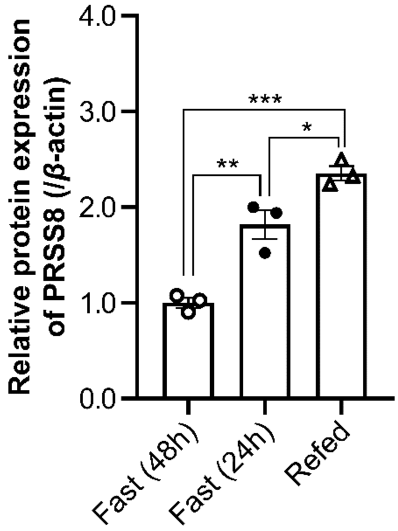


**Figure S16.** Relative protein levels for western blotting in Figure 6b (n=3/group). All data are presented as the mean ± SEM (error bars). *, $p < 0.05$; **, $p < 0.01$; ***, $p < 0.001$.

Supplementary Figure S17.

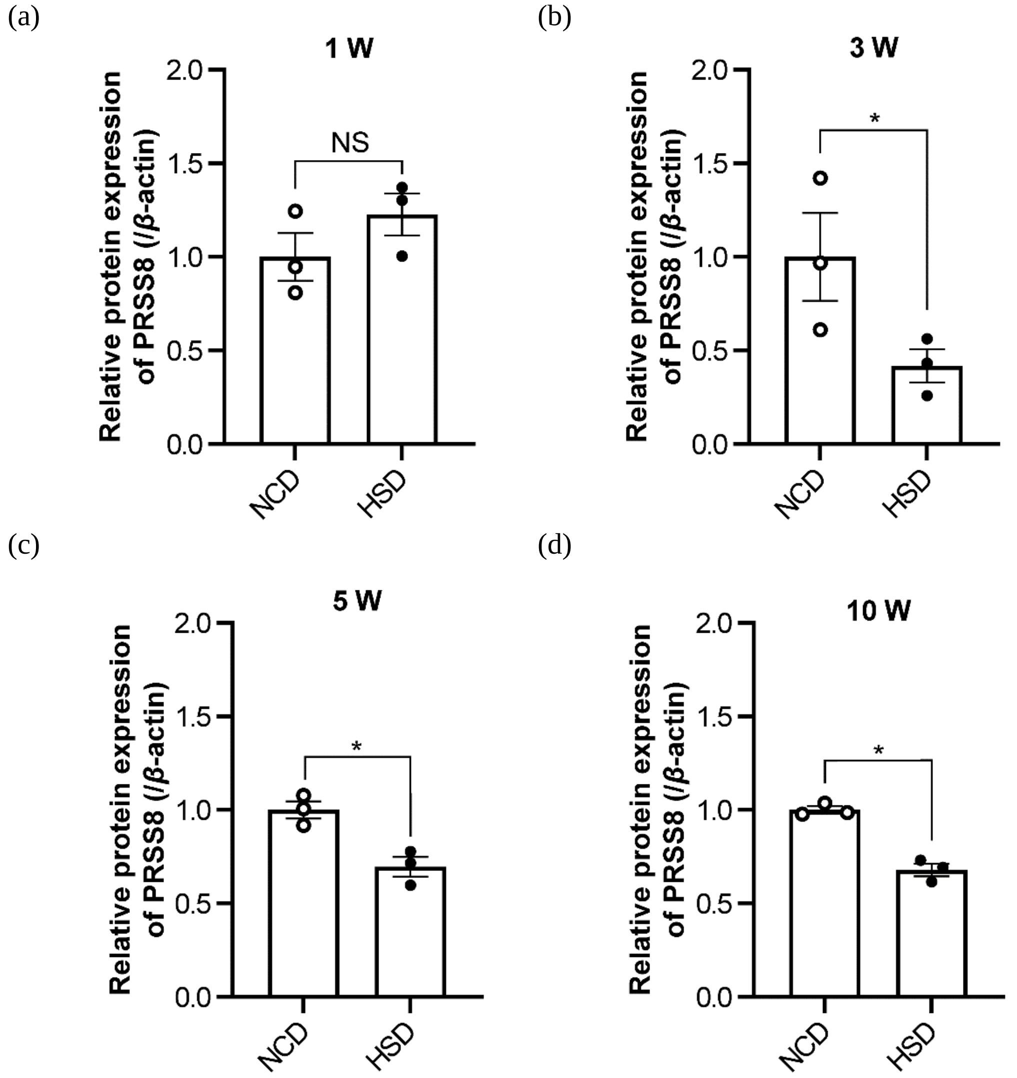


**Figure S17.** Relative protein levels for western blotting in Figure 6c (n=3/group). (a) 1W. (b) 3W. (c) 5W. (d) 10W. All data are presented as the mean ± SEM (error bars). NS, not significant; *, $p < 0.05$.

Supplementary Figure S18.

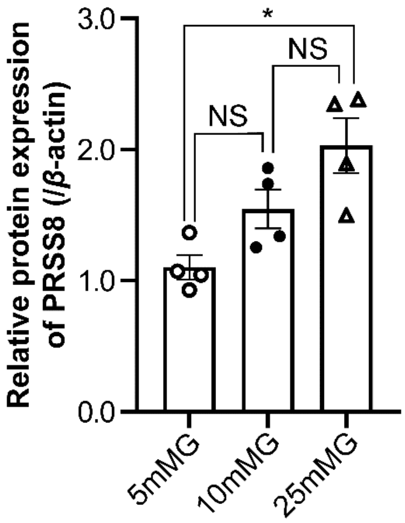


**Figure S18.** Relative protein levels for western blotting in Figure 6d (n=4/group). All data are presented as the mean ± SEM (error bars). NS, not significant; *, $p < 0.05$.

Supplementary Figure S19.

(a)

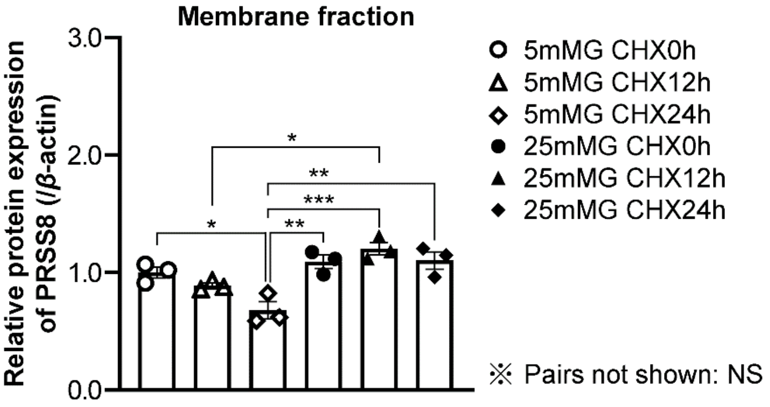


(b)

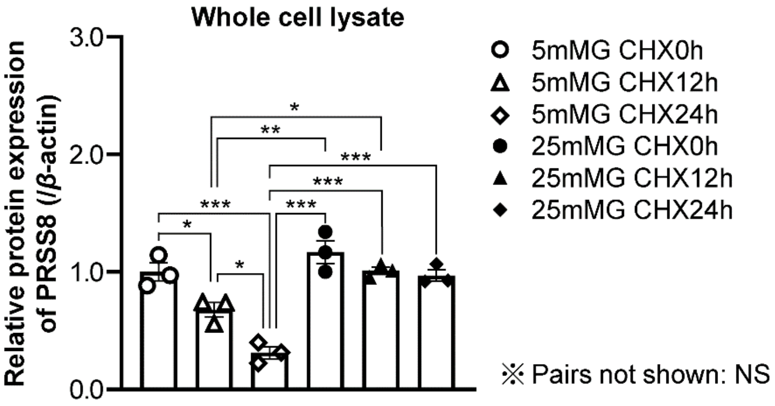


**Figure S19.** Relative protein levels for western blotting in Figure 6f (n=3/group). (a) Membrane fraction. (b) Whole cell lysate. All data are presented as the mean ± SEM (error bars). NS, not significant; *, $p < 0.05$; **, $p < 0.01$; ***, $p < 0.001$.

Supplementary Figure S20.

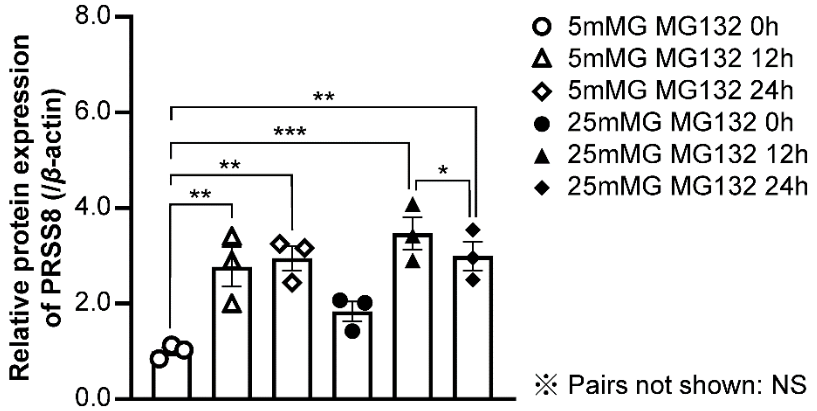


**Figure S20.** Relative protein levels for western blotting in Figure 6g (n=3/group). All data are presented as the mean ± SEM (error bars). NS, not significant; *, $p < 0.05$; **, $p < 0.01$; ***, $p < 0.001$.
